# Supplementary material for: Dietary intake and associated risk factors among pregnant women in Mbeya, Tanzania
Source: PLOS Glob Public Health. 2024 Jan 5;4(1):e0002529. doi: 10.1371/journal.pgph.0002529 (PMC10769095; doi:10.1371/journal.pgph.0002529)
Supplement: Attachment — Submitted filename: PGPH-D-23-01270_R1_reviewer (1).pdf [file pgph.0002529.s004.pdf]

PLOS Global Public Health

Dietary intake and associated risk factors among pregnant women in Mbeya, Tanzania

--Manuscript Draft--

**Manuscript Number:**	PGPH-D-23-01270R1
**Article Type:**	Research Article
**Full Title:**	Dietary intake and associated risk factors among pregnant women in Mbeya, Tanzania
**Short Title:**	Dietary intake among pregnant women
**Corresponding Author:**	Ray Mrisho Masumo, Ph.D, MPH, DDS<br>Tanzania Food and Nutrition Centre<br>Dar es Salaam, Dar es Salaam TANZANIA, UNITED REPUBLIC OF
**Order of Authors:**	Erick Killel
	Geofrey Mchau, Ph.D
	Hamida Mbilikila
	Kaunara Azizi
	Nyamizi Ngasa
	Adam Hancy
	Tedson Lukindo
	Ramadhan Mwiru, MD, Ph.D
	Ramadhan Noor, MD, Ph.D
	Abraham Sanga
	Patrick Codjia
	Germana H. Leyna, MD, Ph.D
	Ray Mrisho Masumo, Ph.D, MPH, DDS
**Keywords:**	Dietary intake; diet quality; pregnancy; marital status
**Abstract:**	Introduction<br><br>Poor dietary intake among pregnant women has seriously detrimental consequences for pregnancy and born children both in developed and developing countries. This study aimed at assessing poor dietary intake among pregnant women and its associated risk factors.<br><br>Methods<br><br>A cross-sectional study design was conducted in Mbeya, Tanzania with a sample size of 420 pregnant women attending antenatal clinics to assess the factors associated with poor dietary intake. Dietary intake was assessed using a piloted questionnaire of the Prime Diet Quality Score. A tested standard questionnaire was also used to collect factors that are associated with poor dietary intake among pregnant women. The strengths of the associations between the dependent and independent variables were tested using the Pearson chi-square tests and the multivariate log-binomial regression method was performed to calculate the adjusted risk ratios (ARR) and 95% confidence interval (CI).<br><br>Results<br><br>The study revealed that out of 420 pregnant women who participated in this study only 12.6%, 29.3%, and 48% consumed at least four servings of fruits, vegetables and refined grains per week respectively. Poor dietary intakes were less likely among cohabiting pregnant women [Adjusted RR 0.22 (95% CI 0.09- 0.50)] and; those who reported taking Fansidar tablets during the pregnancy [Adjusted RR 0.55 (95% CI

Powered by Editorial Manager® and ProduXion Manager® from Aries Systems Corporation

	0.31- 0.96)]. Further, we found that poor dietary intakes were more likely among pregnant women who were classified as overweight and obese by the MUAC above 33cm [Adjusted RR 3.49 (95% CI 1.10- 11.06)].<br><br>Conclusions<br><br>The study results affirm that cohabitation and obesity affect dietary intakes among pregnant women differently compared to married women in rural settings of Tanzania. Further research is needed to investigate the social aspects that link poor dietary intake outcomes for developing a tailored gestational intervention to improve maternal and birth outcomes in sub-Saharan African countries.
**Additional Information:**	
**Question**	**Response**
**Financial Disclosure**<br><br>Enter a financial disclosure statement that describes the sources of funding for the work included in this submission and the role the funder(s) played. This includes grants and any commercial funding of the work or authors.<br><br>This statement will be typeset if the manuscript is accepted for publication.<br><br>*Please review the submission guidelines and the instructions link below for detailed requirements and guidance.*	This research received no specific financial support
**Competing Interests**<br><br>On behalf of all authors, disclose any competing interests that could be perceived to bias this work.<br><br>This statement will be typeset if the manuscript is accepted for publication.<br><br>*Please review the instructions link below and PLOS Global Public Health's competing interests policy to determine what information must be disclosed at submission.*	The authors declare that they have no competing interests
**Data Availability**	All datasets underlying this study are freely available at the public repository https://osf.io/7ysb9/.

Powered by Editorial Manager® and ProduXion Manager® from Aries Systems Corporation

Before publication, Authors are required to make fully available and without restriction all data underlying their findings. Please see our PLOS Data Policy page for detailed information on this policy.<br><br>A **Data Availability Statement**, detailing where the data can be accessed, is required at first submission. Insert your Data Availability Statement in the box below.<br><br>Please see the data reporting section of our submission guidelines for instructions on what you need to include in your Data Availability Statement.<br><br>This statement will be typeset if the manuscript is accepted for publication.<br><br>*PLOS allows rare exemptions to address legal and ethical concerns. If you have legal or ethical restrictions, please use the box below to detail these in full sentences for the Journal team to consider.*	

Powered by Editorial Manager® and ProduXion Manager® from Aries Systems Corporation

Manuscript
Click here to access/download;Manuscript;Dietary intake_FINAL02.doc

Title: Factors associated with poor dietary intake among pregnant women in Mbeya, Tanzania

Authors: Erick Killel[1], Geofrey Mchau[1], Hamida Mbilikila[1], Kaunara Azizi[1], Nyamizi Ngasa[1], Adam Hancy[1], Tedson Lukindo[1], Ramadhan Mwiru[3], Ramadhan Noor[3], Abraham Sanga[3], Patrick Codjia[3], Germana H. Leyna[1, 2] and Ray M. Masumo[1,4]

Author affiliations:

1. Department of Community Health and Nutrition, Tanzania Food and Nutrition Centre, Dar es Salaam, Tanzania
2. Department of Epidemiology and Biostatistics, Muhimbili University of Health and Allied Sciences, Dar es Salaam, Tanzania
3. The United Nations Children's Fund (UNICEF), Dar es Salaam, Tanzania
4. Department of Statistics, University of Dar es Salaam (UDSM), Dar es Salaam, Tanzania

Corresponding author
Dr. Ray M. Masumo, Ph. D.
Email: rmasumo@yahoo.com

Abstract

Introduction: Poor dietary intake among pregnant women has seriously detrimental consequences for pregnancy and born children both in developed and developing countries. This study aimed at assessing poor dietary intake among pregnant women and its associated risk factors.

Methods: A cross-sectional study design was conducted in Mbeya, Tanzania with a sample size of 420 pregnant women attending antenatal clinics to assess the factors associated with poor dietary intake. Dietary intake was assessed using a piloted questionnaire of the Prime Diet Quality Score. A tested standard questionnaire was also used to collect factors that are associated with poor dietary intake among pregnant women. The strengths of the associations between the dependent and independent variables were tested using the Pearson chi-square tests and the multivariate log-binomial regression method was performed to calculate the adjusted risk ratios (ARR) and 95% confidence interval (CI).

Results: The study revealed that out of 420 pregnant women who participated in this study only 12.6%, 29.3%, and 48% consumed at least four servings of fruits, vegetables and refined grains per week respectively. Poor dietary intakes were less likely among cohabiting pregnant women [Adjusted RR 0.22 (95% CI 0.09- 0.50)] and; those who reported taking Fansidar tablets during the pregnancy [Adjusted RR 0.55 (95% CI 0.31- 0.96)]. Further, we found that poor dietary intakes were more likely among pregnant women who were classified as overweight and obese by the MUAC above 33cm [Adjusted RR 3.49 (95% CI 1.10- 11.06)].

Conclusions: The study results affirm that cohabitation and obesity affect dietary intakes among pregnant women differently compared to married women in rural settings of Tanzania. Further research is needed to investigate the social aspects that link poor dietary intake outcomes for developing a tailored gestational intervention to improve maternal and birth outcomes in sub-Saharan African countries.

Keywords: dietary intake; diet quality; pregnancy; marital status

Introduction

Poor dietary intake among pregnant women has seriously detrimental consequences for pregnancy and born children both in developed and developing countries [1]. In 2017, Pelletier and colleagues defined dietary intake and diet quality as one which is hygienically safe, nutritious, balanced and well adapted to the needs of individuals in order to prevent disease, ensuring a good state of health, as well as proper development [2]. Previous studies have consistently reported that the Mediterranean diet is one of the most effective diets in reducing the risk of cardiovascular diseases and overall mortality due to non-communicable diseases [3, 4]. The Mediterranean diet is characterized by a high intake of fish, olive oil, non-starchy vegetables, legumes, whole grains (cereals), fruits, and nuts, as well as a lower intake of dairy products, red and processed meat and a moderate intake of wine [3,4]. Worldwide dietary guidelines vary and each country adapts to suit its specific needs [37]. The dietary guidelines of the United Kingdom and the American 2020-2025 recommended the consumption of at least two portions of fruit and 3 portions of vegetables a day [24, 38].

A Lancet study conducted in 195 countries on the health effects of dietary risks published that in most of the countries, the intake of healthy food such as whole grains, vegetables and fruits were much less compared to unhealthy foods such as processed foods and soft drinks [5]. An epidemiological study from South Africa by Venter and Winterbach revealed a higher dietary intake of fats than the recommended among mid-adolescents [6]. Evidence emanated from Bahrain consistently reported poor consumption of healthy food items compared to unhealthy food items [8]. There are a growing number of studies on dietary intake among pregnant women in many countries, especially industrialised countries [5, 37], however, published research regarding dietary intake among pregnant women in Tanzania is minimal. While the results from

studies based in other countries provide relevant information related to this subject [6], these results cannot be entirely relatable to pregnant women in Tanzania. A recent published longitudinal study in Dar es Salaam, Tanzania among HIV-negative pregnant women reported high consumption of green leafy vegetables and refined grains [22]. Inconsistent findings were reported in another prospective cohort study among 432 pregnant women in the rural settings of Ethiopia where the consumption of vegetables and fruits was poor and associated with a higher risk of adverse pregnancy outcomes [21]. The research work in Tanzania [22] was performed in the urban settings and may not represent the dietary intake in the rural settings. The two previous National Nutrition Surveys in Tanzania, TNNS of 2014 and 2018 lack detailed information on dietary intake and diet quality [7]. Therefore, further high-quality prospective cohort studies are required in Tanzania to enhance the generalisability of the results and help inform policies and programmes.

Dietary intake among pregnant women is affected by various factors, such as socio-demographic and economic status, nutrition status, environmental, cultural, and political [9-13]. Poor dietary intake is nothing new to sub-Saharan African countries but what is of great concern is the fact that it is one of the issues on which a lot of resources have been spent over a period of time with very limited results. The current pieces of literature provide limited information on dietary intake among pregnant women in sub-Saharan African countries because the requirements that would enhance the collection and use of those data, including the use of new technology, in these countries rarely exist [14]. Hence, the present study aimed at examining the factors associated with poor dietary intake among pregnant women in the Mbeya region, Tanzania. The Findings of this study would be a valuable step in developing a tailored gestational intervention to improve maternal and birth outcomes in sub-Saharan African countries.

Methods

Study design

A cross-sectional study design was conducted among pregnant women in seven districts of Mbeya region in Tanzania. The study was carried out from 15th September to 10th December 2022.

Study area

Mbeya region has a population of 2,204,543 (1,068,615 male and 1,135,928 female) and 557,574 women of reproductive age [15]. The total deliveries in the Mbeya region in 2020 were 72,076. There are 17 hospitals, 23 health centres and 278 dispensaries, where 251 health facilities provide reproductive and child health services. This study was conducted at 42 Reproductive and Child Health (RCH) Clinics in seven (7) districts of Mbeya region. The selected RCH clinics in this study are estimated to provide services to approximately 1036 pregnant women [15].

Study Population

All pregnant women aged between 15 to 49 years, less than 28 weeks of gestation, and who attended antenatal visits in Mbeya were invited to participate in the study. A total of 574 pregnant women were invited and 420 (response rate of 73%) agreed to participate. Pregnant women who refused to consent or who were unable to communicate due to illness and, those who were taking medication were excluded from this study.

Sample size and Sampling Procedure

A sample size (n = 420) was considered sufficient based on the Lwanga and Lemeshow formula [16]. Prior to carrying out the study, the proportion of women of reproductive age with poor dietary intake was estimated to be 45%, with a margin error of 5%, a confidence level of 95%, and a design effect of 1.5. Another 10% was added to the sample size to account for non-

responses. The sampling procedure involved two steps [16], a list of 251 governmental and faith-based health facilities providing antenatal services in Mbeya region was obtained and used in a random selection of the health facilities from each district based on probability proportional to size sampling. A total of 42 facilities from a pool of 251 were randomly selected for the survey. An additional two reserved clusters were included in the survey. Given the sampling frame of public health facilities in Mbeya, the probability proportional to size was performed to allocate the number of facilities per district for inclusion in the survey. Therefore, a total of 44 health facilities offering antenatal services located in the Mbeya region were visited and surveyed [16].

Data Collection

Dietary Intake and Diet Quality Assessment

Poor dietary intake and diet quality was assessed by the Prime Diet Quality Score (PDQS) has been developed using a modified Prime Screen questionnaire as a means to characterize diet quality and in the USA, the questionnaire was found to predict factors associated with the lower risk of coronary heart disease (CHD) in a large population [17]. The PDQS contains 21 food groups; 13 are healthy food and, seven (7) are unhealthy food. The PDQS was assessed using 24-hour recalls, which reflected the feeding practice from the previous morning to the morning of the interview [17]. For this study, the PDQS was translated into Kiswahili the main language in Tanzania, spoken proficiently by almost 95% of the population. In the translation process, two translators with different backgrounds independently translated the original questionnaire into Kiswahili. The IMAN project staffs in the field reviewed for semantic, experiential and conceptual equivalence to the original version. Sensitivity to culture and selection of appropriate words were considered. The Kiswahili version of the questionnaire was then given to a translator fluent in both English and Kiswahili to translate back into the original language. This translator

was not shown the original English version. Lastly, all translations and the original questionnaire were given to IMAN project staff in the field in order to consolidate all the versions of the questionnaire and achieve equivalence between the original and target version. Both Kiswahili version and the original English version of the PDQS were administered to 20 female secondary school teachers in Dar es Salaam in two sessions separated by an interval of two weeks to evaluate the quality of the translations in terms of comprehensibility, readability and relevance on face validity and, correlations between the two administrations were calculated.

Participants were asked 'From when you woke up yesterday till you woke up this morning did you consume the following food items: dark green leafy vegetables, cruciferous vegetables, dark orange vegetables and fruits, other vegetables, citrus fruits, other fruits, legumes, nuts and seeds, poultry, fish, whole grains, vegetable liquid oils, white roots and tubers, red meat as a main dish, processed meats, refined grains and baked products, sugar-sweetened beverages, fried foods away from home, sweets, ice cream and low-fat dairy?' Responses were given on a 5- point likert scale; 0= not at all, 1= once, 2= twice, 3= thrice, and 4= fourth or more. Each occasion of food group consumption was considered as a serving. The mean number of servings was computed over the available recall days for each participant. The mean number of servings for each food group was multiplied by 7 to standardise to the number of servings per week, from which points for each food group could be assigned based on whether the food was categorised as healthy or unhealthy [17]. Points were assigned for consumption of healthy food groups as follows: 0–1 serving/week, 0 points; 2–3 servings/week, 1 point; and ≥4 servings/week, 2 points. Scoring for unhealthy food groups was assigned as follows: 0–1 serving/week, 2 points; 2–3 servings/week, 1 point; and ≥4 servings/week, 0 points [17].

Demographic and socio-economic factors

The demographic factors were assessed by asking pregnant women attending antenatal services to provide the following information: Age, marital status, education level, and occupation status. Socio-economic status was assessed by household ownership of durable assets (such as ownership of a car, motorcycle, bicycle, cart, refrigerator, television, radio, etc.), housing characteristics (such as the material of dwelling floor and roof, toilet facilities, etc.), and access to basic services (such as electricity supply, source of drinking water). Household asset data uses simple questions and therefore suffers from less recall or social desirability bias.

Maternal nutrition status was assessed by measuring weight and height. Weight was measured by the nearest 0.1 kg with a battery-powered electronic scale (Seca, Hamburg, Germany), and height was measured to the nearest 0.1 cm with a height model recommended by UNICEF. Height was measured when pregnant women were not wearing shoes or a head covering. Further, Mid Upper Arm Circumference (MUAC) assessed by MUAC tapes was used to assess the nutrition status of pregnant women.

Laboratory assessment

A trained nurse collected blood samples through vein puncture from consented participants. Blood samples were taken into ethylenediaminetetraacetic acid (EDTA) and non-anticoagulated whole blood vacutainers (Becton Dickenson, NJ, USA). Approximately 6mL of venous blood sample was collected on each vacutainer and protected from light. Whole-blood vacutainers were maintained at 4–8°C for less than 2 hours before being transported to the temporary laboratories. Malaria was tested by rapid diagnostic test (SD Bioline, Rep. of Korea), and hemoglobin level was measured by HemoCue HB 201+ analyser (Hemo Cue, Angelholm, Sweden). Assessment of C-reactive protein (CRP), and alpha-1 acid glycoprotein (AGP) was performed with Roche Cobas Integra 400 Plus analyser (Roche Diagnostics GmbH, German). Hemoglobin levels <12.0

and <8.0 g/dL were used to characterise anaemia and severe anaemia, respectively. Serum C-reactive protein (CRP) and Alpha-1-acid glycoprotein (AGP) values of CRP > 5.0 mg/L and AGP > 1.0 g/L respectively were characterized as high inflammatory marks [18].

Data analysis

The data were analysed by using SPSS version 25. The poor dietary intake as dependent variable was assessed as a categorical variable splitting at the median i.e. 0 = good dietary intake and diet quality and, 1= poor dietary intake and diet quality.

An asset-based approach to measuring household socio-economic status is considered an alternative to income and consumption expenditure in low-income countries. Principal Components Analysis (PCA) is a method for determining wealth indices [19]. In this study, household wealth index was assessed as (1) “available and in working condition” or (0) “not available and/or not in working condition” of durable assets, housing characteristics and access to basic services. For constructing a wealth index among pregnant women in Mbeya, the first principal component was used to categorise households into two approximate group’s i.e. lowest and highest group. The strengths of the associations between the dependent and independent variables in bivariate analysis were tested using the Pearson chi-square tests because all variables were categorical and, Log binomial regression method was used first for adjusting confounders and second to identify independent predictors of poor dietary intake and diet quality among the study population. All tests were two-tailed and the significance level was set at 5%.

Ethics Statement

The survey was approved by the Tanzania Ethics Committees i.e. the National Institute for Medical Research with the reference number SZEC-24239/R.A/V.1/151. Date of issue 12th August 2022. All eligible subjects were informed of the purpose and nature of the survey and

those who agreed to participate were asked to sign a written informed consent form. Moreover, a written informed consent was also obtained from the parent/guardian of each participant under 18 years of age. All procedures followed were per the ethical standards of the Helsinki Declaration of 1975 including the confidentiality and, authors had no access to information that could identify individual participants during or after data collection.

Results

Reliability

The internal consistency reliability scales were examined using Cronbach's alpha. Test-retest reliability analysis was performed using kappa statistics and Intra class correlation coefficients (ICC). The agreement between the interviewers and the gold standard on the dietary intake and diet quality assessments on the English and Kiswahili versions were Cohen's kappa of 0.62 and 0.67 respectively. During the field, duplicate interviews were performed randomly with 20 pregnant women. Test-retest reliability of reports on the dietary intake and diet quality assessment using a Kiswahili version in terms of ICC was 0.72 (95% CI 0.64 – 0.78). Thus, acceptable levels of intra-interviews agreement (kappa >0.60) were obtained [20].

The characteristics of the study population

Study participants had a mean age of 25.49 ± 6.37 years. T**able 1** depicts the characteristics of the study population. More than half of the participants were 15-24 years old, about seventy-two percent had completed at least primary education and, 84.3% were self-employed. Five percent (n=21) of pregnant women had Mid Upper Arm Circumference (MUAC) of above 33cm and falls in the category of overweight and obesity. Nine percent (n=38) of pregnant women had serum C- reactive protein (CRP) above 5mg/L and, 19% (n=80) had Alpha-1-acid glycoprotein

(AGP) above 1 g/L. For the construction of wealth index: One-third of the participants lived in houses with electricity; 72% had access to improved sources of drinking water and 65% were not sharing toilet facilities. The pit latrine without washable was the most common type of toilet 31.6 % (n=133). About half (51.5%) used cement as the material of the dwelling floor and 7.8% used thatch/palm leaf as material for the roof. Furthermore, less than two percent (n=6) and 2.4% (n=10) of the participants owned a motor vehicle and a set of television respectively (not in Table 1). Following the PCA analysis, 140 pregnant women (33.4%) fell under the category of lower socio-economic status as shown in Table 1

Dietary intake and diet quality

Table 2 shows the patterns and distribution of dietary intake and diet quality among pregnant women according to PDQS on healthy and unhealthy food. About less than one-third of study participants (n=123) reported consuming more than four times dark green leafy green vegetables, about fifteen percent reported consuming other vegetables, and 57.2% edible vegetable liquid oil per week. Slightly similar trends for other healthy foods, including cruciferous vegetables, dark orange vegetables and fruits, whole citrus fruits, other whole fruits, legumes, nuts and seeds, poultry, fish, whole grains, white roots and tubers and low-fat dairy were also consumed infrequently by pregnant women. Refined grains, baked goods and sugar-sweetened beverages were the most commonly consumed unhealthy foods.

Bivariate analysis

Table 3 depicts the bivariate analysis; poor dietary intake and diet quality were significantly associated with the marital status of pregnant women, and those who received Fansidar tablets during pregnancy. However, the age group of pregnant women, their educational level, occupation status, household wealth index, received iron and folic acid supplements, Serum C-

reactive protein (CRP) and Alpha-1-acid glycoprotein (AGP) were not significantly associated with poor dietary intake and diet quality among pregnant women (p>0.05).

Multivariate analysis

Table 4 presented all variables which were significant (p>0.7) at bivariate analysis and hence qualify to be included in the multivariate analysis. Using a Log binomial regression method, the study found out that poor dietary intake and diet quality were less likely among cohabiting pregnant women [Adjusted RR 0.22 (95% CI 0.09- 0.50)] and; those who reported taking Fansidar (Sulfadoxine and Pyrimethamine, SP) tablets during pregnancy in Mbeya region [Adjusted RR 0.55 (95% CI 0.31- 0.96)]. Further, the study found that poor dietary intake and diet quality were more likely among pregnant women who were classified as overweight and obese by the MUAC [Adjusted RR 3.49 (95% CI 1.10- 11.06)] and; slightly significant among pregnant women of age group 25-29 years old [Adjusted RR 2.05 (95% CI 0.98- 4.29)]. Poor dietary intake and diet quality were not associated with higher concentrations of inflammatory factors i.e. CRP and AGP (p>0.05).

Discussion

This study contributes to our understanding on socio-demographic drivers for poor dietary intake among pregnant women legally married or cohabiting in Tanzania. Poor dietary intake has seriously detrimental consequences for pregnancy and born children both in developed and developing countries [1, 21]. This study found that dietary intake among pregnant women in the rural settings of Tanzania was largely characterised by low intakes of fruits, grains and vegetables. The findings are very similar to those found in Ethiopia [21], Iran [30], and India [31]. However, a study from the urban settings of Tanzania reported a high consumption of vegetables among HIV-negative pregnant women [22]. This could be explained by the difference

in research methodology especially the inclusion and exclusion criteria of the study participants. Evidence shows that, the nutrients in fruits and vegetables such as fibers, vitamins, minerals, and phytochemicals play a key role in human health and well-being [23, 25, 26, 27]. Tanzania has recently developed its national food based dietary guidelines and, it has not yet been fully operationalized. Though other countries like UK and USA their guidelines recommended a plant-based diet, rich in fruits, vegetables, whole grains, and legumes to lower the risk of heart disease, type 2 diabetes, obesity, and other health conditions [24].

In this study, we highlight the factors that affect the dietary intake of pregnant women in the rural settings of Tanzania. Our findings affirmed previous researches on the relationship between cohabitation and dietary intake among pregnant women [29]. Pregnant women who engaged in cohabiting relationships seemed to have better education and financial position and, thus reflected on their decision power over their wealth [29]. From the anecdotal evidence, it is known that cohabiting relationships are a common practice in the study area. According to the study of Dinour and colleagues in 2012, they found that marital status is one of strong socio-demographic factors that greatly influence health-related behaviours and outcomes [28]. However, studies emanating from sub-Saharan African countries merge cohabitation and marriage into one category of marital status and, this is because pregnant women are reluctant to report the status of cohabitation because of stigma [32]. This study managed to assess separate the marriage and cohabitation statuses because there are often different outcomes for the health and well-being of pregnant women and their children in different settings. Further prospective cohort research is needed to investigate the social aspects that link marital transition and dietary intake outcomes among pregnant women in sub-Saharan African countries.

Parallel with globalisation, pronounced changes in the human behaviour and lifestyle such as decreased consumption of fruits and vegetables and increased consumption of unhealthy foods [32], have resulted in escalating rates of overweight and obesity among pregnant women. The trends of overweight and obesity among pregnant women in Tanzania has changed from being considered as a mild disorder to the major causes of morbidity and mortality associated with non-communicable diseases [7]. This study revealed that out of 420 pregnant women who participated in this study, only 12.6% and 29.3% consumed at least four servings of fruits and vegetables per week respectively. Similar findings were documented in the previous studies from low-income countries, where overweight and obese pregnant women consumed less vegetable and fruits [21, 34]. Further, our analysis revealed that pregnant women who were overweight or obese (MUAC measures above 33 cm) were significantly associated with poor dietary intake. Pregnant women with overweight and obesity need nutrition counseling that are supported by scientific evidence and that can be easily understood and translated into everyday life to improve maternal and birth outcomes. Finding from a large, long-term randomised controlled trials provide convincing evidence that changes made in physical activity levels and dietary habits are effective in delaying, and possibly preventing, progression from overweight and obesity to non-communicable diseases [39].

The World Health Organisation (WHO) recommended Intermittent Presumptive Treatment (IPT) to pregnant women to prevent the adverse effects of malaria during pregnant. This study also highlighted important finding, the protective effect of poor dietary intake among pregnant women who reported taking SP tablets (IPT) during their pregnancy. In Tanzania, it is mandatory to offer SP to all pregnant women attending antenatal clinics between 16 and 24 weeks for the first IPT and, between 28 and 32 weeks for the second IPT [40]. The increase in percentage of

pregnant women who use SP for IPT and, protected from poor dietary intake, possibly due to the fact that majority of the study population made early antenatal booking, availability of integrated nutrition and malaria intervention and, a result of continued national advocacy and sensitisation carried out in the country by various stakeholders [41]. Further studies are needed to broaden the understanding on this relationship and, help researchers in sub-Saharan African countries to develop tailored interventions to improve maternal and birth outcomes.

Strengths and limitations

There are several strengths of this study. First, the study was able to use reliable survey data and blood samples from a large population sample and measured diet during the pregnancy using both the Prime Diet Quality Score tool and 24-hour dietary recall. Second, this study provides important information on factors associated with poor dietary intake among pregnant women of gestation period less than 28 weeks, which is important for fetal development, given the rapid cell growth, and development of immune cells and organs in the first trimester [35, 36]. Third, the present study is that it links social and biological data with dietary data and allows analysis of dietary intake. However, there were several limitations of the study. First, we inevitably have some level of measurement error in both dietary and social and biological data, as both were based on self-report. This source of error is, however, expected to be largely random, producing valid estimates for the study population. Second, we derived PDQS scores from 24-hour recalls, and there were limited precedents in published literature for converting these scores to equivalent scores for the food frequency questionnaire (FFQ). The validity of using the PDQS score assessed using 24-hour recall is an area of active research. Notably, the 24-hour recall method is used widely in developing countries and our findings provide support for the use of this metric for deriving PDQS in these settings. Our findings may not be generalisable to populations where

dietary patterns and determinants outcomes differ from rural Tanzania. Associations may be stronger in populations with more prevalent micronutrients and other deficiencies in pregnant women.

Conclusions

The results of this study affirm that cohabitation and obesity affect dietary intake among pregnant women differently compared to marriage in rural settings of Tanzania. Further, the findings suggest that public health action is needed to promote consumption of fruits and vegetables among pregnant women in Mbeya. We recommend prospective cohort research to investigate the social aspects that link poor dietary intake outcomes for developing a tailored gestational intervention to improve maternal and birth outcomes in sub-Saharan African countries.

List of Abbreviation

MUAC: mean upper arm circumference
PDQS: Prime diet quality score
RCH: Reproductive and Child Health
SDG: Sustainable Development Goals
TNNS: National Nutrition Survey

Declarations:

Institutional Review Board Statement

The protocol and data collection procedures for this survey were approved by the Tanzania Ethics Committees i.e. the National Institute for Medical Research with the reference number SZEC-24239/R.A/V.1/151.

Informed Consent Statement

All participants were asked to provide written consent after being read a document emphasizing the voluntary nature of the survey. The risks and benefits of the study were clearly explained to the participants before signing the consent. Also participants were informed of their rights to refuse to participate in the study or withdraw from it at any time, without consequences. All procedures followed were per the ethical standards of the Helsinki Declaration of 1975 including the confidentiality.

Data Availability Statement

All datasets underlying this study are freely available at the public repository https://osf.io/7ysb9/.

Competing interests

The authors declare that they have no competing interests.

Funding

This research received no specific financial support

Authors' contributions

Conceptualization, EK, HM, AH, KA, NN, TL, GM, RN, RM, AS, RN, PC, GHL and RMM; project administration and resources, RMM and GHL; formal analysis and writing—original draft, EK, HM, AH, KA, NN, TL, GM, RN, RM, AS and RN; writing—review and editing, EK, HM, AH, KA, NN, TL, GM, RN, RM, AS, RN, PC, GHL and RMM. All authors have read and agreed to the published version of the manuscript.

Reference

1. Latal-Hajnal B, von Siebenthal K, Kovari H, Bucher HU, Largo RH. Postnatal growth in VLBW infants: significant association with neurodevelopmental outcome. The Journal of pediatrics. 2003;143:163-70.
2. Pelletier JE, Laska MN, MacLehose R, Nelson TF, Nanney MS. Evidence-based policies on school nutrition and physical education: Associations with state-level collaboration, obesity, and socio-economic indicators. Preventive medicine. 2017;99:87-93.
3. Ahmad S, Moorthy MV, Demler OV, Hu FB, Ridker PM, Chasman DI, et al. Assessment of Risk Factors and Biomarkers Associated With Risk of Cardiovascular Disease Among Women Consuming a Mediterranean Diet. JAMA Network Open. 2018;1:e185708.
4. Lopez-Garcia E, Rodriguez-Artalejo F, Li TY, Fung TT, Li S, Willett WC, et al. The Mediterranean-style dietary pattern and mortality among men and women with cardiovascular disease. AJCN. 2013;99:172-80.
5. Afshin A, Sur PJ, Fay KA, Cornaby L, Ferrara G, Salama JS, et al. Health effects of dietary risks in 195 countries, 1990–2017: a systematic analysis for the Global Burden of Disease Study 2017. The lancet. 2019;393:1958-72.
6. Venter IM, Winterbach A. Dietary fat knowledge and intake of mid-adolescents attending public schools in the Bellville/Durbanville area of the city of Cape Town. South African Journal of Clinical Nutrition. 2010;23.
7. Tanzania National Nutrition Survey, TNNS, 2014 final report. Tanzania Food and Nutrition Centre (TFNC), Ministry of Health and Social Welfare. 2015, Dar es Salaam. 2018.
8. Gharib N, Rasheed P. Energy and macronutrient intake and dietary pattern among school children in Bahrain: A cross-sectional study. Nutr J. 2011;10:1-12.
9. Bhanbhro S, Kamal T, Diyo RW, Lipoeto NI, Soltani H. Factors affecting maternal nutrition and health: A qualitative study in a matrilineal community in Indonesia. Plos one. 2020;15:e0234545.
10. Popkin, B.M., Synthesis and implications: C hina's nutrition transition in the context of changes across other low- and middle- income countries. Obesity reviews. 2014;15:60-67.
11. Mayen AL, Marques-Vidal P, Paccaud F, Bovet P, Stringhini S. Socioeconomic determinants of dietary patterns in low-and middle-income countries: a systematic review. The American journal of clinical nutrition. 2014;100:1520-31.
12. Azizan NA, Thangiah N, Su TT, Majid HA. Does a low-income urban population practise healthy dietary habits?. International health. 2018;10:108-15.
13. Gicevic S, Gaskins AJ, Fung TT, Rosner B, Sabanovic E, Milesevic J, et al. Demographic and socio-economic predictors of diet quality among adults in Bosnia and Herzegovina. Public health nutrition. 2019;22:3107-17.
14. Coates JC, Colaiezzi BA, Bell W, Charrondiere UR, Leclercq C. Overcoming dietary assessment challenges in low-income countries: technological solutions proposed by the International Dietary Data Expansion (INDDEX) Project. Nutrients. 2017;9:289.
15. Tanzania National Bureau of Statistics, NBS 2013. Population and housing census 2012.

16. Lwanga SK, Lemeshow S, World Health Organization. Sample size determination in health studies: a practical manual. World Health Organization; 1991. (Accessed on January 2022. URL: https://apps.who.int/iris/handle/10665/40062).
17. Fung TT, Isanaka S, Hu FB, Willett WC. International food group–based diet quality and risk of coronary heart disease in men and women. The American journal of clinical nutrition. 2018;107:120-9.
18. World Health Organization. (2020). WHO guideline on use of ferritin concentrations to assess iron status in individuals and populations. World Health Organization. URL: https://apps.who.int/iris/handle/10665/331505 (Accessed June 2023)
19. Vyas S, Kumaranayake L: Constructing socio-economic status indices: how to use principal components analysis. Health Policy Plan. 2006, 21: 459-468.
20. Landis JR, Koch GG: An application of hierarchical kappa-type statistics in the assessment of majority agreement among multiple observers. Biometrics 1977, 33:363–374.
21. Zerfu TA, Pinto E, Baye K. Consumption of dairy, fruits and dark green leafy vegetables is associated with lower risk of adverse pregnancy outcomes (APO): a prospective cohort study in rural Ethiopia. Nutr Diabetes. 2018;8:52.
22. Madzorera I, Isanaka S, Wang M, Msamanga GI, Urassa W, Hertzmark E, et al. Maternal dietary diversity and dietary quality scores in relation to adverse birth outcomes in Tanzanian women. The American journal of clinical nutrition. 2020;112:695-706.
23. Slavin JL, Lloyd B. Health benefits of fruits and vegetables. Adv Nutr. 2012;3:506-16.
24. Thompson HJ. The dietary guidelines for Americans (2020–2025): pulses, dietary fiber, and chronic disease risk—a call for clarity and action. Nutrients. 2021;13:4034.
25. King, J.C., Physiology of pregnancy and nutrient metabolism. The American journal of clinical nutrition. 2000;**71**:1218S-1225S.
26. Kind KL, Moore VM, Davies MJ. Diet around conception and during pregnancy–effects on fetal and neonatal outcomes. Reproductive biomedicine online. 2006;12:532-41.
27. Imdad A, Bhutta ZA. Nutritional management of the low birth weight/preterm infant in community settings: a perspective from the developing world. The Journal of pediatrics. 2013;162:S107-14.
28. Dinour L, Leung MM, Tripicchio G, Khan S and MC Yeh The Association between Marital Transitions, Body Mass Index, and Weight: A Review of the Literature. J. Obes. 2012; 2012.
29. Dallmann D, Marquis GS, Colecraft EK, Dodoo ND. Marital transition is associated with food insecurity, low dietary diversity, and overweight in a female population in rural Ghana. African Journal of Food, Agriculture, Nutrition and Development. 2023;23:22149-71.
30. Akbari Z, Mansourian M, Kelishadi R. Relationship of the intake of different food groups by pregnant mothers with the birth weight and gestational age: Need for public and individual educational programs. Journal of education and health promotion. 2015;4.
31. Rao S, Yajnik CS, Kanade A, Fall CH, Margetts BM, Jackson AA, et al. Intake of micronutrient-rich foods in rural Indian mothers is associated with the size of their babies at birth: Pune Maternal Nutrition Study. The Journal of nutrition. 2001;131:1217-24.
32. Marston M, Slaymaker E, Cremin I, Floyd S, McGrath N, Kasamba I, et al. Trends in marriage and time spent single in sub-Saharan Africa: a comparative analysis of six

population-based cohort studies and nine Demographic and Health Surveys. Sexually transmitted infections. 2009;85:i64-71.

33. Stuebe AM, Oken E, Gillman MW. Associations of diet and physical activity during pregnancy with risk for excessive gestational weight gain. American journal of obstetrics and gynecology. 2009;201:58-e1.
34. George GC, Milani TJ, Hanss-Nuss H, Freeland-Graves JH. Compliance with dietary guidelines and relationship to psychosocial factors in low-income women in late postpartum. Journal of the American Dietetic Association. 2005 Jun 1;105(6):916-26.
35. Palmer AC. Nutritionally mediated programming of the developing immune system. Advances in nutrition. 2011;2:377-95.
36. Gruszfeld D, Socha P. Early nutrition and health: short-and long-term outcomes. InEvidence-Based Research in Pediatric Nutrition. Karger Publishers.2013;108: 32-39.
37. World Health Organisation, Food Based Dietary Guidelines in the WHO European Region. 2003. URL: http://www.euro.who.int (Accessed June 2023)
38. Culliford AE, Bradbury J, Medici EB. Improving Communication of the UK Sustainable Healthy Dietary Guidelines the Eatwell Guide: A Rapid Review. Sustainability. 2023;15:6149.
39. Harris SB, Petrella RJ and Leadbetter W. Lifestyle interventions for type II diabetes relevance for clinical practice. Canadian Family Physician. 2003;49:1618-1625.
40. Falade CO, Yusuf BO, Fadero FF, Mokuolu O, Hamer DH, Salako L. Intermittent preventive treatment with sulphadoxine-pyrimethamine is effective in preventing maternal and placental malaria in Ibadan, south-western Nigeria. Malaria J. 2007;6:1–8.
41. Mubyazi GM, Bygbjerg IC, Magnussen P, Olsen O, Byskov J, Hansen KS, et al. Prospects, achievements, challenges and opportunities for scaling-up malaria chemoprevention in pregnancy in Tanzania: the perspective of national level officers. Malaria J. 2008;135:1–6.

LIST OF TABLES

Table 1: Frequency distribution of the socio-demographic and economic characteristics of pregnant women in Mbeya (n=420)

Variable	Categories	Frequency (n)	Percentage (%)
Age group (Years)	15-19	82	19.5
	20-24	133	31.6
	25-29	99	23.6
	35+	106	25.2
Education	No education	34	8.1
	Primary	301	71.7
	Secondary and above	85	20.2
Marital status	Married	238	56.5
	Cohabit	133	31.6
	Single/ Divorced	49	11.6
Number of pregnancies	Primigravida	104	24.7
	Multigravida	316	75.1
Trimester of pregnancy	First trimester (<12 weeks)	109	26.0
	Second trimester (12-26 weeks)	311	74.0
Received of iron and folic acid supplements	No	155	36.8
	Yes	265	62.9
Received Fansidar (SP) during pregnancy	No	204	48.5
	Yes	216	51.3
Occupation status	Formal employment	15	3.6
	Self employed	355	84.3
	Not employed	51	12.1
Household wealth Index	Higher socio-economic	140	33.4
	Middle socio-economic	139	33.2
	lower socio-economic	140	33.4
Mid Upper Arm Circumference (MUAC)	Thin (<23cm)	16	3.8
	Normal (between 23 and	383	91.2

	33cm)		
	Overweight or obesity (above 33 cm)	21	5.0
Malaria infection	No	402	95.7
	Yes	18	4.3
Serum C- reactive protein (CRP)	CRP≤5mg/L	383	91.0
	CRP>5mg/L	38	9.0
Alpha-1-acid glycoprotein (AGP)	AGP<=1 g/L	341	81.0
	AGP>1 g/L	80	19.0

Table 2: The patterns and distribution of food groups consumption among pregnant women according to PDQS score (n=420)

**Healthy foods**			
Serving per week	***0–1 serving/week n (%)***	***2–3 servings/week n (%)***	***≥4 servings/week n (%)***
Dark leafy green vegetables	142(33.7)	156 (37.1)	123 (29.2)
Cruciferous vegetables	393(93.4)	20(4.8)	8(1.9)
Dark orange vegetables and fruits	263(62.5)	105(24.9)	53(12.6)
Other vegetables	266(63.2)	93 (22.1)	62(14.7)
Whole citrus fruits	391 (92.9)	25 (5.9)	5 (1.2)
Other whole fruits	308(73.2)	78(18.5)	35(8.3)
Legumes	259 (61.5)	104 (24.7)	58(13.9)
Nuts and seeds	275(65.3)	107(25.4)	39(9.3)
Poultry	389(92.4)	21(5.0)	11(2.6)
Fish	277(65.8)	98(23.3)	46(10.9)

Whole grains	324(77.0)	68(16.1)	29(6.9)
Vegetable liquid oils	46(10.9)	134(31.8)	241(57.2)
White roots and tubers	189(44.9)	161(38.2)	71(16.9)
Low fat diary	349(82.9)	56(13.3)	16(3.8)
**Unhealthy foods**			
Serving per week	***0–1 serving/week n (%)***	***2–3 servings/week n (%)***	***≥4 servings/week n (%)***
Red meats	311(73.9)	85(20.2)	25(5.9)
Sweets and ice cream	349(82.9)	58 (13.8)	14 (3.3)
Fried foods obtained away from Home	348(82.7)	65(15.4)	8(1.9)
Processed meat	412(97.9)	8(1.9)	1(0.2)
Refined grains and baked goods	74(17.6)	145(34.4)	202 (48.0)
Sugar sweetened beverages	249(59.1)	146(34.7)	26(6.2)

Table 3: Bivariate analysis on the factors associated with poor dietary quality among pregnant women in Mbeya (n=420): **Chi square test**

**Variable**	**Categories**	**Good dietary quality**	**Poor dietary quality**	**P-value**
		% (n)	***% (n)***	
Age group (Years)	15-19	16.8 (30)	21.6 (52)	0.129
	20-24	30.7 (55)	32.4 (78)	
	25-29	29.1 (52)	19.5 (47)	
	35+	23.5 (42)	26.6 (64)	
Education	No education	8.3 (15)	7.9 (19)	0.696
	Primary	70.6 (127)	72.6 (175)	
	Secondary and above	21.1 (38)	19.5 (47)	
Marital status	Married	70.0 (126)	46.5 (112)	0.000
	Cohabit	17.8 (32)	42.3 (102)	
	Single/ Divorced	12.2 (22)	11.2 (27)	

Trimester of pregnancy	First trimester (<12 weeks)	27.8 (50)	24.9 (60)	0.506
	Second trimester (12-26 weeks)	72,2 (130)	75.1 (181)	
Received of iron and folic acid supplements	No	38.5 (69)	35.7 (86)	0.548
	Yes	61.5 (110)	64.3(155)	
Received Fansidar during pregnancy	No	43.6 (78)	52.3 (126)	0.077
	Yes	56.4 (101)	47.7 (115)	
Occupation status	Formal employment	5.6 (10)	2.1 (5)	0.156
	Self employed	83.3 (150)	85.5 (206)	
	Not employed	11.1 (20)	12.4 (30)	
Household wealth Index	Higher socio-economic	33.0 (59)	34.0 (82)	0.365
	Middle socio-economic	30.2 (54)	35.3 (85)	
	lower socio-economic	36.9 (66)	30.7 (74)	
Mid Upper Arm Circumference (MUAC)	Thin (<23cm)	3.9 (7)	3.7 (9)	0. 079
	Normal (between 23 and 33cm)	91.6 (164)	90.9 (219)	
	Overweight or obesity (above 33 cm)	4.5 (8)	5.4 (13)	
Malaria infection	No	96.7 (174)	95.0 (229)	0.409
	Yes	3.3 (6)	5.0 (12)	
Serum C-reactive protein (CRP)	CRP≤5mg/L	89.4 (161)	92.1 (222)	0.344
	CRP >5mg/L	50.0 (19)	7.9 (19)	
Alpha-1-acid glycoprotein (AGP)	AGP<=1 g/L	80.6 (145)	81.3 (196)	0.542
	AGP>1 g/L	19.4 (35)	18.7 (45)	

Table 4: Multivariate Log binomial regression methods were used to assess factors associated with poor dietary intake among pregnant women in Mbeya (n=420).

			95% confidence interval for Adj. RR	
**Variable**	**Category**	**Adjusted RR**	**Lower bound**	**Upper bound**
**Age group**	15-19	1.22	0.51	2.89
	20-24	1.31	0.66	2.59
	25-29	**2.05**	**0.98**	**4.29**
	35+	1	1	1
Marital status	Married	1.35	0.60	3.04
	Cohabit	**0.22**	**0.09**	**0.50**
	Single/divorced	1	1	1
Occupational status	Formal employment	4.29	0.83	22.17
	Self employed	1.02	0.45	2.29
	Not employed	1	1	1
Education level	No formal	0.73	0.21	2.56
	Primary	0.95	0.47	1.91
	Secondary and above	1	1	1
Household wealth index	Higher socio-economic	1.40	0.77	2.56
	Middle socio-economic	0.70	0.36	1.35
	lower socio-economic	1	1	1
Mid Upper Arm Circumference (MUAC)	Thin (<23cm)	3.02	0.52	17.50
	Overweight or obesity (above 33 cm)	**3.49**	**1.10**	**11.06**
	Normal (between 23 and 33cm)	1	1	1
Taken Fansidar during this pregnancy	Yes	**0.55**	**0.31**	**0.96**
	No	1	1	1
Malaria status	Yes	1.89	0.54	6.62
	No	1	1	1
Received of iron and folic acid supplements	Yes	1.09	0.62	1.90

	No	1	1	1
Alpha-1-acid glycoprotein (AGP)	AGP<=1 g/L	0.94	0.48	1.81
	AGP>1 g/L	1	1	1
Serum C- reactive protein (CRP)	CRP≤5mg/L	0.60	0.24	1.50
	CRP>5mg/L	1	1	1
Number of pregnancy	Primigravida	1.09	0.54	2.22
	Multigravida	1	1	1
Trimester of pregnancy	First trimester (<12 weeks)	1.14	0.64	2.03
	Second trimester (12-26 weeks)	1	1	1

The reference group is last category

Manuscript revised clean
Click here to access/download;Manuscript;Dietary intake_FINAL02_05_10_2023_clean.doc

Title: Dietary intake and associated risk factors among pregnant women in Mbeya, Tanzania

Authors: Erick Killel[1], Geofrey Mchau[1], Hamida Mbilikila[1], Kaunara Azizi[1], Nyamizi Ngasa[1], Adam Hancy[1], Tedson Lukindo[1], Ramadhan Mwiru[3], Ramadhan Noor[3], Abraham Sanga[3], Patrick Codjia[3], Germana H. Leyna[1, 2] and Ray M. Masumo[1,4]

Author affiliations:

1. Department of Community Health and Nutrition, Tanzania Food and Nutrition Centre, Dar es Salaam, Tanzania
2. Department of Epidemiology and Biostatistics, Muhimbili University of Health and Allied Sciences, Dar es Salaam, Tanzania
3. The United Nations Children's Fund (UNICEF), Dar es Salaam, Tanzania
4. Department of Statistics, University of Dar es Salaam (UDSM), Dar es Salaam, Tanzania

Corresponding author
Dr. Ray M. Masumo, Ph. D.
Email: rmasumo@yahoo.com

Abstract

Introduction: Poor dietary intake among pregnant women has serious detrimental consequences for pregnancy and offspring both in developed and developing countries. This study aimed to assess dietary intake and associated risk factors among pregnant women.

Methods: A cross-sectional study was conducted in Mbeya, Tanzania with a sample size of 420 pregnant women attending antenatal clinics to assess the factors associated with dietary intake. Dietary intake was assessed using a piloted questionnaire of the Prime Diet Quality Score. A tested standard questionnaire was also used to collect factors that are associated with dietary intake among pregnant women. The strengths of the associations between the dependent and independent variables were tested using the Pearson chi-square tests and the multivariate log-binomial regression method was performed to calculate the adjusted risk ratios (ARR) and 95% confidence interval (CI).

Results: The study revealed that out of 420 pregnant women who participated in this study only 12.6% and 29.3% consumed at least four servings of fruits and vegetables per week respectively. Poor dietary intakes were less likely among cohabiting pregnant women [Adjusted RR 0.22 (95% CI 0.09- 0.50)] and; those who reported taking Fansidar tablets during the pregnancy [Adjusted RR 0.55 (95% CI 0.31- 0.96)]. Further, we found that poor dietary intakes were more likely among pregnant women who were classified as overweight and obese by the MUAC above 33cm [Adjusted RR 3.49 (95% CI 1.10- 11.06)].

Conclusions: The study results affirm that cohabitation and obesity affect dietary intakes among pregnant women differently compared to married women in rural settings of Tanzania. Further research is needed to investigate the social aspects that link dietary intake outcomes for developing a tailored gestational intervention to improve maternal and birth outcomes in sub-Saharan African countries.

Keywords: dietary intake; diet quality; pregnancy; marital status

Introduction

Poor dietary intake among pregnant women has serious detrimental consequences for pregnancy and offspring both in developed and developing countries [1]. In 2017, Pelletier and colleagues defined dietary quality as one that is hygienically safe, nutritious, balanced, and well adapted to the needs of individuals in order to prevent disease, ensure a good state of health, as well as proper development [2]. Previous studies have consistently reported that the Mediterranean diet is one of the most effective diets in reducing the risk of cardiovascular diseases and overall mortality due to non-communicable diseases [3, 4]. The Mediterranean diet is characterized by a high intake of fish, olive oil, non-starchy vegetables, legumes, whole grains (cereals), fruits, and nuts, as well as a lower intake of dairy products, red and processed meat and a moderate intake of wine [3,4]. Worldwide dietary guidelines vary and each country adapts to suit its specific needs [37]. The dietary guidelines of the United Kingdom and the American 2020-2025 recommended the consumption of at least two portions of fruit and 3 portions of vegetables a day [24, 38].

A Lancet study conducted in 195 countries on the health effects of dietary risks published that in most of the countries, the intake of healthy food such as whole grains, vegetables and fruits were much less compared to unhealthy foods such as processed foods and soft drinks [5]. An epidemiological study from South Africa by Venter and Winterbach revealed a higher dietary intake of fats than the recommended among mid-adolescents [6]. Evidence emanated from Bahrain consistently reported poor consumption of healthy food items compared to unhealthy food items [8]. There are a growing number of studies on dietary intake among pregnant women in many countries, especially industrialised countries [5, 37], however, published research regarding dietary intake among pregnant women in Tanzania is minimal. While the results from

studies based in other countries provide relevant information related to this subject [6], these results cannot be entirely relatable to pregnant women in Tanzania. A recently published longitudinal study in Dar es Salaam, Tanzania among pregnant women reported high consumption of green leafy vegetables and refined grains [22]. Inconsistent findings were reported in another prospective cohort study among 432 pregnant women in the rural settings of Ethiopia where the consumption of vegetables and fruits was poor and associated with a higher risk of adverse pregnancy outcomes [21]. The research work in Tanzania [22] was performed in urban settings and may not represent the dietary intake in rural settings. The two previous National Nutrition Surveys in Tanzania, TNNS of 2014 and 2018 lack detailed information on dietary intake and diet quality [7]. Therefore, further high-quality prospective cohort studies are required in Tanzania to enhance the generalisability of the results and help inform policies and programmes.

Dietary intake among pregnant women is affected by various factors, such as socio-demographic and economic status, nutrition status, environmental, cultural, and political [9-13]. Adequate dietary intake is nothing new to sub-Saharan African countries but what is of great concern is the fact that it is one of the issues on which a lot of resources have been spent over a period of time with very limited results. The current pieces of literature provide limited information on dietary intake among pregnant women in sub-Saharan African countries because the requirements that would enhance the collection and use of those data, including the use of new technology, in these countries rarely exist [14]. Hence, the present study aimed at examining dietary intake and associated risk factors among pregnant women in the Mbeya region, Tanzania. The findings of this study would be a valuable step in developing a tailored gestational intervention to improve maternal and birth outcomes in sub-Saharan African countries.

Methods

Study design

A cross-sectional study was conducted among pregnant women in seven districts of Mbeya region in Tanzania. The study was carried out from 15th September to 10th December 2022.

Study area

Mbeya region has a population of 2,204,543 (1,068,615 male and 1,135,928 female) and 557,574 women of reproductive age [15]. The total deliveries in the Mbeya region in 2020 were 72,076. There are 17 hospitals, 23 health centres and 278 dispensaries, where 251 health facilities provide reproductive and child health services. This study was conducted at 42 Reproductive and Child Health (RCH) Clinics in seven (7) districts of the Mbeya region. The selected RCH clinics in this study are estimated to provide services to approximately 1036 pregnant women [15].

Study Population

All pregnant women aged between 15 to 49 years, less than 28 weeks of gestation, and who attended antenatal visits in Mbeya were invited to participate in the study. This is according to the minimal risk in research involving pregnant women and offspring [43]. A total of 574 pregnant women were invited and 420 (response rate of 73.0%) agreed to participate. Pregnant women who refused to consent or who were unable to communicate due to illness and, those who were taking medication other than routine medication which are provided in the antenatal care were excluded from this study.

Sample size and Sampling Procedure

A sample size (n = 420) was considered sufficient based on the Lwanga and Lemeshow formula [16]. Prior to carrying out the study, the proportion of women of reproductive age with poor dietary intake was estimated to be 45%, with a margin error of 5%, a confidence level of 95%,

and a design effect of 1.5. Another 10% was added to the sample size to account for non-responses. The sampling procedure involved two steps [16], a list of 251 governmental and faith-based health facilities providing antenatal services in the Mbeya region was obtained and used in a random selection of the health facilities from each district based on probability proportional to size sampling. A total of 42 facilities from a pool of 251 were randomly selected for the survey. An additional two reserved clusters were included in the survey. Given the sampling frame of public health facilities in Mbeya, the probability proportional to size was performed to allocate the number of facilities per district for inclusion in the survey. Therefore, a total of 44 health facilities offering antenatal services located in the Mbeya region were visited and surveyed [16].

Data Collection

Dietary Intake Assessment

Dietary intake was assessed by the Prime Diet Quality Score (PDQS) developed in the USA using a modified Prime Screen questionnaire as a means to characterize diet quality [17]. The questionnaire was first found to predict factors associated with the lower risk of coronary heart disease (CHD) in a large population in the USA [17], and diet quality among adults in Bosnia and Herzegovina [45]. In Tanzania, Yang and colleagues employed the questionnaire in a prospective pregnancy cohort study [22]. The PDQS contains 21 food groups; 13 are healthy food and, seven (7) are unhealthy food. The PDQS was assessed using 24-hour recalls, which reflected the feeding practice from the previous morning to the morning of the interview [17,22,44]. For this study, the questionnaire was translated into Kiswahili the main language in Tanzania, spoken proficiently by almost 95% of the population. In the translation process, two translators with different backgrounds independently translated the original questionnaire into Kiswahili. The IMAN project staff in the field reviewed for semantic, experiential, and

conceptual equivalence to the original version. Sensitivity to culture and selection of appropriate words were considered. The Kiswahili version of the questionnaire was then given to a translator fluent in both English and Kiswahili to translate back into the original language. This translator was not shown the original English version. Lastly, all translations and the original questionnaire were given to IMAN project staff in the field in order to consolidate all the versions of the questionnaire and achieve equivalence between the original and target versions. Both the Kiswahili version and the original English version of the PDQS were administered to 20 female secondary school teachers in Dar es Salaam in two sessions separated by an interval of two weeks to evaluate the quality of the translations in terms of comprehensibility, readability and relevance on face validity and, correlations between the two administrations were calculated. However, a 30-day recall was not a part of this study which might have different comparable outcomes.

Participants were asked ‘From when you woke up yesterday till you woke up this morning did you consume the following food items: dark green leafy vegetables, cruciferous vegetables, dark orange vegetables and fruits, other vegetables, citrus fruits, other fruits, legumes, nuts and seeds, poultry, fish, whole grains, vegetable liquid oils, white roots and tubers, red meat as a main dish, processed meats, refined grains and baked products, sugar-sweetened beverages, fried foods away from home, sweets, ice cream and low-fat dairy?’ Responses were given on a 5-point likert scale; 0= not at all, 1= once, 2= twice, 3= thrice, and 4= fourth or more. Each occasion of food group consumption was considered as a serving. The mean number of servings was computed over the available recall days for each participant. The mean number of servings for each food group was multiplied by 7 to standardise the number of servings per week, from which points for each food group could be assigned based on whether the food was categorised as healthy or

unhealthy [17,22,44]. Points were assigned for consumption of healthy food groups as follows: 0–1 serving/week, 0 points; 2–3 servings/week, 1 point; and ≥4 servings/week, 2 points. Scoring for unhealthy food groups was assigned as follows: 0–1 serving/week, 2 points; 2–3 servings/week, 1 point; and ≥4 servings/week, 0 points [17,22,44].

Demographic and socio-economic factors

The demographic factors were assessed by asking pregnant women attending antenatal services to provide the following information: Age, marital status, education level, and occupation status. Socio-economic status was assessed by household ownership of durable assets (such as ownership of a car, motorcycle, bicycle, cart, refrigerator, television, radio, etc.), housing characteristics (such as the material of dwelling floor and roof, toilet facilities, etc.), and access to basic services (such as electricity supply, source of drinking water). Household asset data uses simple questions and therefore suffers from less recall or social desirability bias.

Anthropometric measurements

Maternal nutrition status was assessed by measuring weight and height. Weight was measured by the nearest 0.1 kg with a battery-powered electronic scale (Seca, Hamburg, Germany), and height was measured to the nearest 0.1 cm with a height model recommended by UNICEF. Height was measured when pregnant women were not wearing shoes or a head covering. Further, Mid Upper Arm Circumference (MUAC) assessed by MUAC tapes was used to assess the nutrition status of pregnant women [42].

Laboratory assessment

A trained nurse collected blood samples through vein puncture from consented participants. Blood samples were taken into ethylenediaminetetraacetic acid (EDTA) and non-anticoagulated whole blood vacutainers (Becton Dickenson, NJ, USA). Approximately 6mL of venous blood

sample was collected on each vacutainer and protected from light. Whole-blood vacutainers were maintained at 4–8°C for less than 2 hours before being transported to the temporary laboratories. Malaria was tested by rapid diagnostic test (SD Bioline, Rep. of Korea), and hemoglobin level was measured by HemoCue HB 201+ analyser (Hemo Cue, Angelholm, Sweden). Assessment of C-reactive protein (CRP), and alpha-1 acid glycoprotein (AGP) was performed with Roche Cobas Integra 400 Plus analyser (Roche Diagnostics GmbH, German). Hemoglobin levels <12.0 and <8.0 g/dL were used to characterise anaemia and severe anaemia, respectively. Serum C-reactive protein (CRP) and Alpha-1-acid glycoprotein (AGP) values of CRP > 5.0 mg/L and AGP > 1.0 g/L respectively were characterized as high inflammatory marks [18].

Data analysis

The data were analysed by using SPSS version 25. The dietary intake as dependent variable was assessed as a categorical variable splitting at the median i.e. 0 = good dietary intake and, 1= poor dietary intake.

An asset-based approach to measuring household socio-economic status is considered an alternative to income and consumption expenditure in low-income countries. Principal Components Analysis (PCA) is a method for determining wealth indices [19]. In this study, household wealth index was assessed as (1) “available and in working condition” or (0) “not available and/or not in working condition” of durable assets, housing characteristics and access to basic services. For constructing a wealth index among pregnant women in Mbeya, the first principal component was used to categorise households into two approximate group’s i.e. lowest and highest group. The strengths of the associations between the dependent and independent variables in bivariate analysis were tested using the Pearson chi-square tests because all variables were categorical. Independent variables that were significant at arbitrary levels in the bivariate

analysis were selected for multivariate analysis. We based this on the Wald test with a P-value cut-off of 0.7. In multivariate analysis, Log binomial regression method were used first for adjusting confounders and second to identify independent predictors of dietary intake among the study population, and the significance level was set at 5%.

Ethics Statement

The survey was approved by the Tanzania Ethics Committees i.e. the National Institute for Medical Research with the reference number SZEC-24239/R.A/V.1/151. Date of issue 12th August 2022. All eligible subjects were informed of the purpose and nature of the survey and those who agreed to participate were asked to sign a written informed consent form. Moreover, a written informed consent was obtained from the parent/guardian of each participant under 18 years of age. All procedures followed were per the ethical standards of the Helsinki Declaration of 1975 including the confidentiality and, authors had no access to information that could identify individual participants during or after data collection.

Results

Reliability

The internal consistency reliability scales were examined using Cronbach's alpha. Test-retest reliability analysis was performed using kappa statistics and Intra class correlation coefficients (ICC). The agreement between the interviewers and the gold standard on the dietary intake assessments on the English and Kiswahili versions were Cohen's kappa of 0.62 and 0.67 respectively. During the field, duplicate interviews were performed randomly with 20 pregnant women. Test-retest reliability of reports on the dietary intake assessment using a Kiswahili

version in terms of ICC was 0.72 (95% CI 0.64 – 0.78). Thus, acceptable levels of intra-interviews agreement (kappa >0.60) were obtained [20].

The characteristics of the study population

Study participants had a mean age of 25.49 ± 6.37 years. T**able 1** depicts the characteristics of the study population. More than half of the participants were 15-24 years old, about seventy-two percent had completed at least primary education and, 84.3% were self-employed. Five percent (n=21) of pregnant women had Mid Upper Arm Circumference (MUAC) of above 33cm and falls in the category of overweight and obesity. Nine percent (n=38) of pregnant women had serum C- reactive protein (CRP) above 5mg/L and, 19% (n=80) had Alpha-1-acid glycoprotein (AGP) above 1 g/L. For the construction of wealth index: One-third of the participants lived in houses with electricity; 72.0% had access to improved sources of drinking water and 65.0% were not sharing toilet facilities. The pit latrine without washable was the most common type of toilet 31.6 % (n=133). About half (51.5%) used cement as the material of the dwelling floor and 7.8% used thatch/palm leaf as material for the roof. Furthermore, less than two percent (n=6) and 2.4% (n=10) of the participants owned a motor vehicle and a set of television respectively (not in Table 1). Following the PCA analysis, 140 pregnant women (33.4%) fell under the category of lower socio-economic status as shown in Table 1

Dietary intake and diet quality

Among 420 pregnant women who participated in the study, two hundred forty (57.2%) fell into the group of poor dietary intake. The median PDQS was 16 (the 25th and 75th percentiles were 14.0 and 18.0, respectively). **Table 2** shows the patterns and distribution of dietary intake among pregnant women according to PDQS on healthy and unhealthy food shows that 57.2 of the study participants consumed more than four servings of edible vegetable liquid oil per week out of the

14 healthy foods assessed. Furthermore, the healthy foods that were less consumed per week were cruciferous vegetables (93.4%), whole citrus fruits (92.9%), and poultry (92.4%). However, refined grains and baked goods was high (48.0%) high serving consumed per week out of the six unhealthy foods assessed. .

Bivariate analysis

Table 3 depicts the bivariate analysis; poor dietary intake were significantly associated with the marital status of pregnant women, and those who received Fansidar tablets during pregnancy ($p>0.05$). However, the age group of pregnant women, their educational level, occupation status, household wealth index, received iron and folic acid supplements, Serum C- reactive protein (CRP) and Alpha-1-acid glycoprotein (AGP) were not significantly associated with poor dietary intake and diet quality among pregnant women ($p>0.05$).

Multivariate analysis

Table 4 presented all variables which were significant at arbitrary levels in the bivariate analysis and hence qualify to be included in the multivariate analysis. Using a Log binomial regression method, the study found out that poor dietary intake were less likely among cohabiting pregnant women [Adjusted RR 0.22 (95% CI 0.09- 0.50)] and; those who reported taking Fansidar (Sulfadoxine and Pyrimethamine, SP) tablets during pregnancy in Mbeya region [Adjusted RR 0.55 (95% CI 0.31- 0.96)]. Further, the study found that poor dietary intake were more likely among pregnant women who were classified as overweight and obese by the MUAC [Adjusted RR 3.49 (95% CI 1.10- 11.06)] and; slightly significant among pregnant women of age group 25-29 years old [Adjusted RR 2.05 (95% CI 0.98- 4.29)]. Poor dietary intake was not associated with higher concentrations of inflammatory factors i.e. CRP and AGP.

Discussion

This study contributes to our understanding on socio-demographic drivers for poor dietary intake among pregnant women legally married or cohabiting in Tanzania. Worldwide, poor dietary intake has negative consequences for pregnancy and born children [1, 21]. This study found that dietary intake among pregnant women in the rural settings of Tanzania was largely characterised by low intakes of fruits and vegetables. The findings are very similar to those found in Ethiopia [21], Iran [30], and India [31]. However, a study from the urban settings of Tanzania reported a high consumption of vegetables among pregnant women [22]. This could be explained by the difference in research methodology especially the inclusion and exclusion criteria of the study participants, and also the difference in dietary assessment tools used between the studies.

Evidence shows that, the nutrients in fruits and vegetables such as fibers, vitamins, minerals, and phytochemicals play a key role in human health and well-being [23, 25, 26, 27]. Tanzania has recently developed its national food based dietary guidelines and, it has not yet been fully operationalized. Though other countries like UK and USA their guidelines recommended a plant-based diet, rich in fruits, vegetables, whole grains, and legumes to lower the risk of heart disease, type 2 diabetes, obesity, and other health conditions [24].

In this study, we highlight the factors that affect the dietary intake of pregnant women in the rural settings of Tanzania. Our findings affirmed previous researches on the relationship between cohabitation and dietary intake among pregnant women [29]. Pregnant women who engaged in cohabiting relationships seemed to have better education and financial position and, thus reflected on their decision power over their wealth [29]. From the anecdotal evidence, it is known that cohabiting relationships are a common practice in the study area. According to the study of Dinour and colleagues in 2012, they found that marital status is one of strong socio-demographic factors that greatly influence health-related behaviours and outcomes [28].

However, studies emanating from sub-Saharan African countries merge cohabitation and marriage into one category of marital status and, this is because pregnant women are reluctant to report the status of cohabitation because of stigma [32]. This study managed to assess separate the marriage and cohabitation statuses because there are often different outcomes for the health and well-being of pregnant women and their children in different settings. Further prospective cohort research is needed to investigate the social aspects that link marital transition and dietary intake outcomes among pregnant women in sub-Saharan African countries.

Parallel with globalisation, pronounced changes in the human behaviour and lifestyle such as decreased consumption of fruits and vegetables and increased consumption of unhealthy foods [32], have resulted in escalating rates of overweight and obesity among pregnant women. The trends of overweight and obesity among pregnant women in Tanzania has changed from being considered as a mild disorder to the major causes of morbidity and mortality associated with non-communicable diseases [7]. According to MUAC measures, this study found that only twenty one pregnant women had MUAC measures above 33cm [42]. Further, our study revealed that out of 420 pregnant women, only 12.6% and 29.3% consumed at least four servings of fruits and vegetables per week respectively. Similar findings were documented in the previous studies from low-income countries, where overweight and obese pregnant women consumed less vegetable and fruits [21, 34]. In multivariate analysis, our data revealed that pregnant women who were overweight or obese were significantly associated with poor dietary intake. Pregnant women with overweight and obesity need nutrition counseling that are supported by scientific evidence and that can be easily understood and translated into everyday life to improve maternal and birth outcomes. Finding from a large, long-term randomised controlled trials provide convincing evidence that changes made in physical activity levels and dietary habits are effective

in delaying, and possibly preventing, progression from overweight and obesity to non-communicable diseases [39]. Future prospective cohort research is needed to investigate the link between obese and dietary patterns among pregnant women.

This study also highlighted important finding, the protective effect to poor dietary intake among pregnant women who reported taking SP tablets. In Tanzania, SP tablets are offered to all pregnant women attending antenatal clinics between 16 and 24 weeks and, between 28 and 32 weeks [40]. The linkage between taking SP tablets and poor dietary intake protection could not established because malaria and nutrition interventions are well integrated into antenatal care in Tanzania, and both impart pregnant women with essential knowledge [41]. Further studies are needed to broaden the understanding on this relationship and, help researchers in sub-Saharan African countries to develop tailored interventions to improve maternal and birth outcomes.

Strengths and limitations

There are several strengths of this study. First, the study was able to use reliable survey data and blood samples from a large population sample and measured diet during the pregnancy using both the Prime Diet Quality Score tool and 24-hour dietary recall. Second, this study provides important information on factors associated with dietary intake among pregnant women of gestation period less than 28 weeks, which is important for fetal development, given the rapid cell growth, and development of immune cells and organs in the first trimester [35, 36]. Third, the present study is that it links social and biological data with dietary data and allows analysis of dietary intake. However, there were several limitations of the study. First, we inevitably have some level of measurement error in both dietary and social and biological data, as both were based on self-report. This source of error is, however, expected to be largely random, producing valid estimates for the study population. Second, we derived PDQS scores from 24-hour recalls,

and there were limited precedents in published literature for converting these scores to equivalent scores for the food frequency questionnaire (FFQ). The validity of using the PDQS score assessed using 24-hour recall is an area of active research. Notably, the 24-hour recall method is used widely in developing countries and our findings provide support for the use of this metric for deriving PDQS in these settings. Our findings may not be generalisable to populations where dietary patterns and determinants outcomes differ from rural Tanzania. Associations may be stronger in populations with more prevalent micronutrients and other deficiencies in pregnant women.

Conclusions

The results of this study affirm that cohabitation and obesity affect dietary intake among pregnant women differently compared to marriage in rural settings of Tanzania. Further, the findings suggest that public health action is needed to promote the consumption of fruits and vegetables among pregnant women in Mbeya. We recommend prospective cohort research to investigate the social aspects that link poor dietary intake outcomes for developing a tailored gestational intervention to improve maternal and birth outcomes in sub-Saharan African countries.

Supporting information
Strobe statement (strobe_statement_dietary.docx)
Copy of ethical clearance (Ethical clearance.pdf)

List of Abbreviation

MUAC: mean upper arm circumference
PDQS: Prime diet quality score
RCH: Reproductive and Child Health
SDG: Sustainable Development Goals
TNNS: National Nutrition Survey

Declarations:

Institutional Review Board Statement

The protocol and data collection procedures for this survey were approved by the Tanzania Ethics Committees i.e. the National Institute for Medical Research with the reference number SZEC-24239/R.A/V.1/151.

Informed Consent Statement

All participants were asked to provide written consent after being read a document emphasizing the voluntary nature of the survey. The risks and benefits of the study were clearly explained to the participants before signing the consent. Also participants were informed of their rights to refuse to participate in the study or withdraw from it at any time, without consequences. All procedures followed were per the ethical standards of the Helsinki Declaration of 1975 including the confidentiality.

Data Availability Statement

All datasets underlying this study are freely available at the public repository https://osf.io/7ysb9/.

Competing interests

The authors declare that they have no competing interests.

Funding

This research received no specific financial support

Authors' contributions

Conceptualization, EK, HM, AH, KA, NN, TL, GM, RN, RM, AS, RN, PC, GHL and RMM; project administration and resources, RMM and GHL; formal analysis and writing—original draft, EK, HM, AH, KA, NN, TL, GM, RN, RM, AS and RN; writing—review and editing, EK, HM, AH, KA, NN, TL, GM, RN, RM, AS, RN, PC, GHL and RMM. All authors have read and agreed to the published version of the manuscript.

Reference

1. Latal-Hajnal B, von Siebenthal K, Kovari H, Bucher HU, Largo RH. Postnatal growth in VLBW infants: significant association with neurodevelopmental outcome. The Journal of pediatrics. 2003;143:163-70.
2. Pelletier JE, Laska MN, MacLehose R, Nelson TF, Nanney MS. Evidence-based policies on school nutrition and physical education: Associations with state-level collaboration, obesity, and socio-economic indicators. Preventive medicine. 2017;99:87-93.
3. Ahmad S, Moorthy MV, Demler OV, Hu FB, Ridker PM, Chasman DI, et al. Assessment of Risk Factors and Biomarkers Associated With Risk of Cardiovascular Disease Among Women Consuming a Mediterranean Diet. JAMA Network Open. 2018;1:e185708.
4. Lopez-Garcia E, Rodriguez-Artalejo F, Li TY, Fung TT, Li S, Willett WC, et al. The Mediterranean-style dietary pattern and mortality among men and women with cardiovascular disease. AJCN. 2013;99:172-80.
5. Afshin A, Sur PJ, Fay KA, Cornaby L, Ferrara G, Salama JS, et al. Health effects of dietary risks in 195 countries, 1990–2017: a systematic analysis for the Global Burden of Disease Study 2017. The lancet. 2019;393:1958-72.
6. Venter IM, Winterbach A. Dietary fat knowledge and intake of mid-adolescents attending public schools in the Bellville/Durbanville area of the city of Cape Town. South African Journal of Clinical Nutrition. 2010;23.
7. Tanzania National Nutrition Survey, TNNS, 2014 final report. Tanzania Food and Nutrition Centre (TFNC), Ministry of Health and Social Welfare. 2015, Dar es Salaam. 2018.
8. Gharib N, Rasheed P. Energy and macronutrient intake and dietary pattern among school children in Bahrain: A cross-sectional study. Nutr J. 2011;10:1-12.
9. Bhanbhro S, Kamal T, Diyo RW, Lipoeto NI, Soltani H. Factors affecting maternal nutrition and health: A qualitative study in a matrilineal community in Indonesia. Plos one. 2020;15:e0234545.
10. Popkin, B.M., Synthesis and implications: C hina's nutrition transition in the context of changes across other low- and middle- income countries. Obesity reviews. 2014;15:60-67.
11. Mayen AL, Marques-Vidal P, Paccaud F, Bovet P, Stringhini S. Socioeconomic determinants of dietary patterns in low-and middle-income countries: a systematic review. The American journal of clinical nutrition. 2014;100:1520-31.
12. Azizan NA, Thangiah N, Su TT, Majid HA. Does a low-income urban population practise healthy dietary habits?. International health. 2018;10:108-15.
13. Gicevic S, Gaskins AJ, Fung TT, Rosner B, Sabanovic E, Milesevic J, et al. Demographic and socio-economic predictors of diet quality among adults in Bosnia and Herzegovina. Public health nutrition. 2019;22:3107-17.
14. Coates JC, Colaiezzi BA, Bell W, Charrondiere UR, Leclercq C. Overcoming dietary assessment challenges in low-income countries: technological solutions proposed by the International Dietary Data Expansion (INDDEX) Project. Nutrients. 2017;9:289.
15. Tanzania National Bureau of Statistics, NBS 2013. Population and housing census 2012.

16. Lwanga SK, Lemeshow S, World Health Organization. Sample size determination in health studies: a practical manual. World Health Organization; 1991. (Accessed on January 2022. URL: https://apps.who.int/iris/handle/10665/40062).
17. Fung TT, Isanaka S, Hu FB, Willett WC. International food group–based diet quality and risk of coronary heart disease in men and women. The American journal of clinical nutrition. 2018;107:120-9.
18. World Health Organization. (2020). WHO guideline on use of ferritin concentrations to assess iron status in individuals and populations. World Health Organization. URL: https://apps.who.int/iris/handle/10665/331505 (Accessed June 2023)
19. Vyas S, Kumaranayake L: Constructing socio-economic status indices: how to use principal components analysis. Health Policy Plan. 2006, 21: 459-468.
20. Landis JR, Koch GG: An application of hierarchical kappa-type statistics in the assessment of majority agreement among multiple observers. Biometrics 1977, 33:363–374.
21. Zerfu TA, Pinto E, Baye K. Consumption of dairy, fruits and dark green leafy vegetables is associated with lower risk of adverse pregnancy outcomes (APO): a prospective cohort study in rural Ethiopia. Nutr Diabetes. 2018;8:52.
22. Madzorera I, Isanaka S, Wang M, Msamanga GI, Urassa W, Hertzmark E, et al. Maternal dietary diversity and dietary quality scores in relation to adverse birth outcomes in Tanzanian women. The American journal of clinical nutrition. 2020;112:695-706.
23. Slavin JL, Lloyd B. Health benefits of fruits and vegetables. Adv Nutr. 2012;3:506-16.
24. Thompson HJ. The dietary guidelines for Americans (2020–2025): pulses, dietary fiber, and chronic disease risk—a call for clarity and action. Nutrients. 2021;13:4034.
25. King, J.C., Physiology of pregnancy and nutrient metabolism. The American journal of clinical nutrition. 2000;**71**:1218S-1225S.
26. Kind KL, Moore VM, Davies MJ. Diet around conception and during pregnancy–effects on fetal and neonatal outcomes. Reproductive biomedicine online. 2006;12:532-41.
27. Imdad A, Bhutta ZA. Nutritional management of the low birth weight/preterm infant in community settings: a perspective from the developing world. The Journal of pediatrics. 2013;162:S107-14.
28. Dinour L, Leung MM, Tripicchio G, Khan S and MC Yeh The Association between Marital Transitions, Body Mass Index, and Weight: A Review of the Literature. J. Obes. 2012; 2012.
29. Dallmann D, Marquis GS, Colecraft EK, Dodoo ND. Marital transition is associated with food insecurity, low dietary diversity, and overweight in a female population in rural Ghana. African Journal of Food, Agriculture, Nutrition and Development. 2023;23:22149-71.
30. Akbari Z, Mansourian M, Kelishadi R. Relationship of the intake of different food groups by pregnant mothers with the birth weight and gestational age: Need for public and individual educational programs. Journal of education and health promotion. 2015;4.
31. Rao S, Yajnik CS, Kanade A, Fall CH, Margetts BM, Jackson AA, et al. Intake of micronutrient-rich foods in rural Indian mothers is associated with the size of their babies at birth: Pune Maternal Nutrition Study. The Journal of nutrition. 2001;131:1217-24.
32. Marston M, Slaymaker E, Cremin I, Floyd S, McGrath N, Kasamba I, et al. Trends in marriage and time spent single in sub-Saharan Africa: a comparative analysis of six

population-based cohort studies and nine Demographic and Health Surveys. Sexually transmitted infections. 2009;85:i64-71.

33. Stuebe AM, Oken E, Gillman MW. Associations of diet and physical activity during pregnancy with risk for excessive gestational weight gain. American journal of obstetrics and gynecology. 2009;201:58-e1.
34. George GC, Milani TJ, Hanss-Nuss H, Freeland-Graves JH. Compliance with dietary guidelines and relationship to psychosocial factors in low-income women in late postpartum. Journal of the American Dietetic Association. 2005 Jun 1;105(6):916-26.
35. Palmer AC. Nutritionally mediated programming of the developing immune system. Advances in nutrition. 2011;2:377-95.
36. Gruszfeld D, Socha P. Early nutrition and health: short-and long-term outcomes. InEvidence-Based Research in Pediatric Nutrition. Karger Publishers.2013;108: 32-39.
37. World Health Organisation, Food Based Dietary Guidelines in the WHO European Region. 2003. URL: http://www.euro.who.int (Accessed June 2023)
38. Culliford AE, Bradbury J, Medici EB. Improving Communication of the UK Sustainable Healthy Dietary Guidelines the Eatwell Guide: A Rapid Review. Sustainability. 2023;15:6149.
39. Harris SB, Petrella RJ and Leadbetter W. Lifestyle interventions for type II diabetes relevance for clinical practice. Canadian Family Physician. 2003;49:1618-1625.
40. Falade CO, Yusuf BO, Fadero FF, Mokuolu O, Hamer DH, Salako L. Intermittent preventive treatment with sulphadoxine-pyrimethamine is effective in preventing maternal and placental malaria in Ibadan, south-western Nigeria. Malaria J. 2007;6:1–8.
41. Mubyazi GM, Bygbjerg IC, Magnussen P, Olsen O, Byskov J, Hansen KS, et al. Prospects, achievements, challenges and opportunities for scaling-up malaria chemoprevention in pregnancy in Tanzania: the perspective of national level officers. Malaria J. 2008;135:1–6.
42. Okereke CE, Anyaehie UB, Dim CC, Iyare EE, Nwagha UI. Evaluation of some anthropometric indices for the diagnosis of obesity in pregnancy in Nigeria: a cross-sectional study. African health sciences. 2013;13:1034-40.
43. Strong C. Minimal risk in research involving pregnant women and fetuses. J Law Med Ethics. 2011;39:529-38.
44. Logan D, McEvoy CT, McKenna G, Kee F, Linden G, Woodside JV. Association between oral health status and future dietary intake and diet quality in older men: The PRIME study. Journal of dentistry. 2020;92:103265.
45. Gicevic S, Gaskins AJ, Fung TT, Rosner B, Sabanovic E, Milesevic J, et al. Demographic and socio-economic predictors of diet quality among adults in Bosnia and Herzegovina. Public health nutrition. 2019;22:3107-17.

LIST OF TABLES

Table 1: Frequency distribution of the socio-demographic and economic characteristics of pregnant women in Mbeya (n=420)

Variable	Categories	Frequency (n)	Percentage (%)
Age group (Years)	15-19	82	19.5
	20-24	133	31.6
	25-29	99	23.6
	35+	106	25.2
Education	No education	34	8.1
	Primary	301	71.7
	Secondary and above	85	20.2
Marital status	Married	238	56.5
	Cohabit	133	31.6
	Single/ Divorced	49	11.6
Number of pregnancies	Primigravida	104	24.7
	Multigravida	316	75.1
Trimester of pregnancy	First trimester (<12 weeks)	109	26.0
	Second trimester (12-26 weeks)	311	74.0
Received of iron and folic acid supplements	No	155	36.8
	Yes	265	62.9
Received Fansidar (SP) during pregnancy	No	204	48.5
	Yes	216	51.3
Occupation status	Formal employment	15	3.6
	Self employed	355	84.3
	Not employed	51	12.1
Household wealth Index	Higher socio-economic	140	33.4
	Middle socio-economic	139	33.2
	lower socio-economic	140	33.4
Mid Upper Arm Circumference (MUAC)	Thin (<23cm)	16	3.8
	Normal (between 23 and	383	91.2

	33cm)		
	Overweight or obesity (above 33 cm)	21	5.0
Malaria infection	No	402	95.7
	Yes	18	4.3
Serum C- reactive protein (CRP)	CRP≤5mg/L	383	91.0
	CRP>5mg/L	38	9.0
Alpha-1-acid glycoprotein (AGP)	AGP<=1 g/L	341	81.0
	AGP>1 g/L	80	19.0

Table 2: The patterns and distribution of food groups consumption among pregnant women according to PDQS score (n=420)

**Healthy foods**			
Serving per week	***0–1 serving/week n (%)***	***2–3 servings/week n (%)***	***≥4 servings/week n (%)***
Dark leafy green vegetables	142(33.7)	156 (37.1)	123 (29.2)
Cruciferous vegetables	393(93.4)	20(4.8)	8(1.9)
Dark orange vegetables and fruits	263(62.5)	105(24.9)	53(12.6)
Other vegetables	266(63.2)	93 (22.1)	62(14.7)
Whole citrus fruits	391 (92.9)	25 (5.9)	5 (1.2)
Other whole fruits	308(73.2)	78(18.5)	35(8.3)
Legumes	259 (61.5)	104 (24.7)	58(13.9)
Nuts and seeds	275(65.3)	107(25.4)	39(9.3)
Poultry	389(92.4)	21(5.0)	11(2.6)
Fish	277(65.8)	98(23.3)	46(10.9)
Whole grains	324(77.0)	68(16.1)	29(6.9)
Vegetable liquid oils	46(10.9)	134(31.8)	241(57.2)
White roots and tubers	189(44.9)	161(38.2)	71(16.9)
Low fat diary	349(82.9)	56(13.3)	16(3.8)
**Unhealthy foods**			
Serving per week	***0–1 serving/week n (%)***	***2–3 servings/week n (%)***	***≥4 servings/week n (%)***
Red meats	311(73.9)	85(20.2)	25(5.9)
Sweets and ice cream	349(82.9)	58 (13.8)	14 (3.3)
Fried foods obtained away from Home	348(82.7)	65(15.4)	8(1.9)
Processed meat	412(97.9)	8(1.9)	1(0.2)
Refined grains and baked goods	74(17.6)	145(34.4)	202 (48.0)
Sugar sweetened beverages	249(59.1)	146(34.7)	26(6.2)

Table 3: Bivariate analysis on the factors associated with poor dietary quality among pregnant women in Mbeya (n=420): Chi square test

Variable	Categories	Good dietary quality	Poor dietary quality	P-value
		% (n)	*% (n)*	
Age group (Years)	15-19	16.8 (30)	21.6 (52)	0.129
	20-24	30.7 (55)	32.4 (78)	
	25-29	29.1 (52)	19.5 (47)	
	35+	23.5 (42)	26.6 (64)	
Education	No education	8.3 (15)	7.9 (19)	0.696
	Primary	70.6 (127)	72.6 (175)	
	Secondary and above	21.1 (38)	19.5 (47)	
Marital status	Married	70.0 (126)	46.5 (112)	0.000
	Cohabit	17.8 (32)	42.3 (102)	
	Single/ Divorced	12.2 (22)	11.2 (27)	
Trimester of pregnancy	First trimester (<12 weeks)	27.8 (50)	24.9 (60)	0.506
	Second trimester (12-26 weeks)	72,2 (130)	75.1 (181)	
Received of iron and folic acid supplements	No	38.5 (69)	35.7 (86)	0.548
	Yes	61.5 (110)	64.3(155)	
Received Fansidar during pregnancy	No	43.6 (78)	52.3 (126)	0.077
	Yes	56.4 (101)	47.7 (115)	
Occupation status	Formal employment	5.6 (10)	2.1 (5)	0.156
	Self employed	83.3 (150)	85.5 (206)	
	Not employed	11.1 (20)	12.4 (30)	
Household wealth Index	Higher socio-economic	33.0 (59)	34.0 (82)	0.365
	Middle socio-economic	30.2 (54)	35.3 (85)	
	lower socio-economic	36.9 (66)	30.7 (74)	

Mid Upper Arm Circumference (MUAC)	Thin (<23cm)	3.9 (7)	3.7 (9)	0. 079
	Normal (between 23 and 33cm)	91.6 (164)	90.9 (219)	
	Overweight or obesity (above 33 cm)	4.5 (8)	5.4 (13)	
Malaria infection	No	96.7 (174)	95.0 (229)	0.409
	Yes	3.3 (6)	5.0 (12)	
Serum C-reactive protein (CRP)	CRP≤5mg/L	89.4 (161)	92.1 (222)	0.344
	CRP >5mg/L	50.0 (19)	7.9 (19)	
Alpha-1-acid glycoprotein (AGP)	AGP<=1 g/L	80.6 (145)	81.3 (196)	0.542
	AGP>1 g/L	19.4 (35)	18.7 (45)	

Table 4: Multivariate Log binomial regression methods were used to assess factors associated with poor dietary intake among pregnant women in Mbeya (n=420).

			**95% confidence interval for Adj. RR**	
**Variable**	**Category**	**Adjusted RR**	**Lower bound**	**Upper bound**
**Age group**	15-19	1.22	0.51	2.89
	20-24	1.31	0.66	2.59
	25-29	**2.05**	**0.98**	**4.29**
	35+	1	1	1
Marital status	Married	1.35	0.60	3.04
	Cohabit	**0.22**	**0.09**	**0.50**
	Single/divorced	1	1	1
Occupational status	Formal employment	4.29	0.83	22.17
	Self employed	1.02	0.45	2.29
	Not employed	1	1	1
Education level	No formal	0.73	0.21	2.56

	Primary	0.95	0.47	1.91
	Secondary and above	1	1	1
Household wealth index	Higher socio-economic	1.40	0.77	2.56
	Middle socio-economic	0.70	0.36	1.35
	lower socio-economic	1	1	1
Mid Upper Arm Circumference (MUAC)	Thin (<23cm)	3.02	0.52	17.50
	Overweight or obesity (above 33 cm)	**3.49**	**1.10**	**11.06**
	Normal (between 23 and 33cm)	1	1	1
Taken Fansidar during this pregnancy	Yes	**0.55**	**0.31**	**0.96**
	No	1	1	1
Malaria status	Yes	1.89	0.54	6.62
	No	1	1	1
Received of iron and folic acid supplements	Yes	1.09	0.62	1.90
	No	1	1	1
Alpha-1-acid glycoprotein (AGP)	AGP<=1 g/L	0.94	0.48	1.81
	AGP>1 g/L	1	1	1
Serum C- reactive protein (CRP)	CRP≤5mg/L	0.60	0.24	1.50
	CRP>5mg/L	1	1	1
Number of pregnancy	Primigravida	1.09	0.54	2.22
	Multigravida	1	1	1
Trimester of pregnancy	First trimester (<12 weeks)	1.14	0.64	2.03
	Second trimester (12-26 weeks)	1	1	1

The reference group is last category

Click here to access/download
Supporting Information
strobe_statement_dietary.docx

Copy of ethical clearance

Click here to access/download
Supporting Information
Ethical clearance.pdf

Title: <u>Dietary intake and </u>~~Factors~~ associated <u>risk factors</u>~~with poor dietary intake~~ among pregnant women in Mbeya, Tanzania

Authors: Erick Killel[1], Geofrey Mchau[1], Hamida Mbilikila[1], Kaunara Azizi[1], Nyamizi Ngasa[1], Adam Hancy[1], Tedson Lukindo[1], Ramadhan Mwiru[3], Ramadhan Noor[3], Abraham Sanga[3], Patrick Codjia[3], Germana H. Leyna[1, 2] and Ray M. Masumo[1,4]

Author affiliations:

1. Department of Community Health and Nutrition, Tanzania Food and Nutrition Centre, Dar es Salaam, Tanzania
2. Department of Epidemiology and Biostatistics, Muhimbili University of Health and Allied Sciences, Dar es Salaam, Tanzania
3. The United Nations Children's Fund (UNICEF), Dar es Salaam, Tanzania
4. Department of Statistics, University of Dar es Salaam (UDSM), Dar es Salaam, Tanzania

Corresponding author
Dr. Ray M. Masumo, Ph. D.
Email: rmasumo@yahoo.com

Abstract

Introduction: Poor dietary intake among pregnant women has serious~~ly~~ detrimental consequences for pregnancy and ~~born children~~offspring both in developed and developing countries. This study aimed ~~at assessing~~to assess ~~poor~~ dietary intake ~~among pregnant women~~ and its associated risk factors~~.~~ among pregnant women.

Methods: A cross-sectional study ~~design~~ was conducted in Mbeya, Tanzania with a sample size of 420 pregnant women attending antenatal clinics to assess the factors associated with poor dietary intake. Dietary intake was assessed using a piloted questionnaire of the Prime Diet Quality Score. A tested standard questionnaire was also used to collect factors that are associated with poor dietary intake among pregnant women. The strengths of the associations between the dependent and independent variables were tested using the Pearson chi-square tests and the multivariate log-binomial regression method was performed to calculate the adjusted risk ratios (ARR) and 95% confidence interval (CI).

Results: The study revealed that out of 420 pregnant women who participated in this study only 12.6%~~,~~ and 29.3% ~~and 48%~~ consumed at least four servings of fruits~~,~~ and vegetables ~~and refined grains~~ per week respectively. Poor dietary intakes were less likely among cohabiting pregnant women [Adjusted RR 0.22 (95% CI 0.09- 0.50)] and; those who reported taking Fansidar tablets during the pregnancy [Adjusted RR 0.55 (95% CI 0.31- 0.96)]. Further, we found that poor dietary intakes were more likely among pregnant women who were classified as overweight and obese by the MUAC above 33cm [Adjusted RR 3.49 (95% CI 1.10- 11.06)].

Conclusions: The study results affirm that cohabitation and obesity affect dietary intakes among pregnant women differently compared to married women in rural settings of Tanzania. Further research is needed to investigate the social aspects that link ~~poor~~ dietary intake outcomes for developing a tailored gestational intervention to improve maternal and birth outcomes in sub-Saharan African countries.

Keywords: dietary intake; diet quality; pregnancy; marital status

Introduction

Poor dietary intake among pregnant women has serious~~ly~~ detrimental consequences for pregnancy and ~~born children~~offspring both in developed and developing countries [1]. In 2017, Pelletier and colleagues defined dietary ~~intake and diet~~ quality as one ~~which~~ that is hygienically safe, nutritious, balanced, and well adapted to the needs of individuals in order to prevent disease, ensure~~ing~~ a good state of health, as well as proper development [2]. Previous studies have consistently reported that the Mediterranean diet is one of the most effective diets in reducing the risk of cardiovascular diseases and overall mortality due to non-communicable diseases [3, 4]. The Mediterranean diet is characterized by a high intake of fish, olive oil, non-starchy vegetables, legumes, whole grains (cereals), fruits, and nuts, as well as a lower intake of dairy products, red and processed meat and a moderate intake of wine [3,4]. Worldwide dietary guidelines vary and each country adapts to suit its specific needs [37]. The dietary guidelines of the United Kingdom and the American 2020-2025 recommended the consumption of at least two portions of fruit and 3 portions of vegetables a day [24, 38].

A Lancet study conducted in 195 countries on the health effects of dietary risks published that in most of the countries, the intake of healthy food such as whole grains, vegetables and fruits were much less compared to unhealthy foods such as processed foods and soft drinks [5]. An epidemiological study from South Africa by Venter and Winterbach revealed a higher dietary intake of fats than the recommended among mid-adolescents [6]. Evidence emanated from Bahrain consistently reported poor consumption of healthy food items compared to unhealthy food items [8]. There are a growing number of studies on dietary intake among pregnant women in many countries, especially industrialised countries [5, 37], however, published research regarding dietary intake among pregnant women in Tanzania is minimal. While the results from

studies based in other countries provide relevant information related to this subject [6], these results cannot be entirely relatable to pregnant women in Tanzania. A recently published longitudinal study in Dar es Salaam, Tanzania among ~~HIV negative~~ pregnant women reported high consumption of green leafy vegetables and refined grains [22]. Inconsistent findings were reported in another prospective cohort study among 432 pregnant women in the rural settings of Ethiopia where the consumption of vegetables and fruits was poor and associated with a higher risk of adverse pregnancy outcomes [21]. The research work in Tanzania [22] was performed in ~~the~~ urban settings and may not represent the dietary intake in ~~the~~ rural settings. The two previous National Nutrition Surveys in Tanzania, TNNS of 2014 and 2018 lack detailed information on dietary intake and diet quality [7]. Therefore, further high-quality prospective cohort studies are required in Tanzania to enhance the generalisability of the results and help inform policies and programmes.

Dietary intake among pregnant women is affected by various factors, such as socio-demographic and economic status, nutrition status, environmental, cultural, and political [9-13]. ~~Poor~~ Adequate dietary intake is nothing new to sub-Saharan African countries but what is of great concern is the fact that it is one of the issues on which a lot of resources have been spent over a period of time with very limited results. The current pieces of literature provide limited information on dietary intake among pregnant women in sub-Saharan African countries because the requirements that would enhance the collection and use of those data, including the use of new technology, in these countries rarely exist [14]. Hence, the present study aimed at examining dietary intake ~~the~~ and associated risk factors ~~associated with poor dietary intake~~ among pregnant women in the Mbeya region, Tanzania. The ~~Findings~~ findings of this study would be a valuable

step in developing a tailored gestational intervention to improve maternal and birth outcomes in sub-Saharan African countries.

Methods

Study design

A cross-sectional study ~~design~~ was conducted among pregnant women in seven districts of Mbeya region in Tanzania. The study was carried out from 15th September to 10th December 2022.

Study area

Mbeya region has a population of 2,204,543 (1,068,615 male and 1,135,928 female) and 557,574 women of reproductive age [15]. The total deliveries in the Mbeya region in 2020 were 72,076. There are 17 hospitals, 23 health centres and 278 dispensaries, where 251 health facilities provide reproductive and child health services. This study was conducted at 42 Reproductive and Child Health (RCH) Clinics in seven (7) districts of the Mbeya region. The selected RCH clinics in this study are estimated to provide services to approximately 1036 pregnant women [15].

Study Population

All pregnant women aged between 15 to 49 years, less than 28 weeks of gestation, and who attended antenatal visits in Mbeya were invited to participate in the study. This is according to the minimal risk in research involving pregnant women and offspring [43]. A total of 574 pregnant women were invited and 420 (response rate of 73.0%) agreed to participate. Pregnant women who refused to consent or who were unable to communicate due to illness and, those who were taking medication other than routine medication which are provided in the antenatal care were excluded from this study.

Sample size and Sampling Procedure

A sample size (n = 420) was considered sufficient based on the Lwanga and Lemeshow formula [16]. Prior to carrying out the study, the proportion of women of reproductive age with poor dietary intake was estimated to be 45%, with a margin error of 5%, a confidence level of 95%, and a design effect of 1.5. Another 10% was added to the sample size to account for non-responses. The sampling procedure involved two steps [16], a list of 251 governmental and faith-based health facilities providing antenatal services in the Mbeya region was obtained and used in a random selection of the health facilities from each district based on probability proportional to size sampling. A total of 42 facilities from a pool of 251 were randomly selected for the survey. An additional two reserved clusters were included in the survey. Given the sampling frame of public health facilities in Mbeya, the probability proportional to size was performed to allocate the number of facilities per district for inclusion in the survey. Therefore, a total of 44 health facilities offering antenatal services located in the Mbeya region were visited and surveyed [16].

Data Collection

Dietary Intake ~~and Diet Quality~~ Assessment

~~Poor dietary~~Dietary intake ~~and diet quality~~ was assessed by the Prime Diet Quality Score (PDQS) ~~has been~~ developed in the USA using a modified Prime Screen questionnaire as a means to characterize diet quality [17]~~and in the USA,~~. ~~the~~The questionnaire was first found to predict factors associated with the lower risk of coronary heart disease (CHD) in a large population in the USA [17], and diet quality among adults in Bosnia and Herzegovina [45]. In Tanzania, Yang and colleagues employed the questionnaire in a prospective pregnancy cohort study [22]. The PDQS contains 21 food groups; 13 are healthy food and, seven (7) are unhealthy food. The PDQS was assessed using 24-hour recalls, which reflected the feeding practice from the previous morning to the morning of the interview [17,22,44]. For this study, the questionnaire~~PDQS~~ was

translated into Kiswahili the main language in Tanzania, spoken proficiently by almost 95% of the population. In the translation process, two translators with different backgrounds independently translated the original questionnaire into Kiswahili. The IMAN project staffs in the field reviewed for semantic, experiential, and conceptual equivalence to the original version. Sensitivity to culture and selection of appropriate words were considered. The Kiswahili version of the questionnaire was then given to a translator fluent in both English and Kiswahili to translate back into the original language. This translator was not shown the original English version. Lastly, all translations and the original questionnaire were given to IMAN project staff in the field in order to consolidate all the versions of the questionnaire and achieve equivalence between the original and target versions. Both the Kiswahili version and the original English version of the PDQS were administered to 20 female secondary school teachers in Dar es Salaam in two sessions separated by an interval of two weeks to evaluate the quality of the translations in terms of comprehensibility, readability and relevance on face validity and, correlations between the two administrations were calculated. However, a 30-day recall was not a part of this study which might have different comparable outcomes.

Participants were asked ‘From when you woke up yesterday till you woke up this morning did you consume the following food items: dark green leafy vegetables, cruciferous vegetables, dark orange vegetables and fruits, other vegetables, citrus fruits, other fruits, legumes, nuts and seeds, poultry, fish, whole grains, vegetable liquid oils, white roots and tubers, red meat as a main dish, processed meats, refined grains and baked products, sugar-sweetened beverages, fried foods away from home, sweets, ice cream and low-fat dairy?’ Responses were given on a 5--point likert scale; 0= not at all, 1= once, 2= twice, 3= thrice, and 4= fourth or more. Each occasion of food group consumption was considered as a serving. The mean number of servings was

computed over the available recall days for each participant. The mean number of servings for each food group was multiplied by 7 to standardise ~~to~~ the number of servings per week, from which points for each food group could be assigned based on whether the food was categorised as healthy or unhealthy [17,22,44]. Points were assigned for consumption of healthy food groups as follows: 0–1 serving/week, 0 points; 2–3 servings/week, 1 point; and ≥4 servings/week, 2 points. Scoring for unhealthy food groups was assigned as follows: 0–1 serving/week, 2 points; 2–3 servings/week, 1 point; and ≥4 servings/week, 0 points [17,22,44].

Demographic and socio-economic factors

The demographic factors were assessed by asking pregnant women attending antenatal services to provide the following information: Age, marital status, education level, and occupation status. Socio-economic status was assessed by household ownership of durable assets (such as ownership of a car, motorcycle, bicycle, cart, refrigerator, television, radio, etc.), housing characteristics (such as the material of dwelling floor and roof, toilet facilities, etc.), and access to basic services (such as electricity supply, source of drinking water). Household asset data uses simple questions and therefore suffers from less recall or social desirability bias.

Anthropometric measurements

Maternal nutrition status was assessed by measuring weight and height. Weight was measured by the nearest 0.1 kg with a battery-powered electronic scale (Seca, Hamburg, Germany), and height was measured to the nearest 0.1 cm with a height model recommended by UNICEF. Height was measured when pregnant women were not wearing shoes or a head covering. Further, Mid Upper Arm Circumference (MUAC) assessed by MUAC tapes was used to assess the nutrition status of pregnant women [42].

Laboratory assessment

A trained nurse collected blood samples through vein puncture from consented participants. Blood samples were taken into ethylenediaminetetraacetic acid (EDTA) and non-anticoagulated whole blood vacutainers (Becton Dickenson, NJ, USA). Approximately 6mL of venous blood sample was collected on each vacutainer and protected from light. Whole-blood vacutainers were maintained at 4–8°C for less than 2 hours before being transported to the temporary laboratories. Malaria was tested by rapid diagnostic test (SD Bioline, Rep. of Korea), and hemoglobin level was measured by HemoCue HB 201+ analyser (Hemo Cue, Angelholm, Sweden). Assessment of C-reactive protein (CRP), and alpha-1 acid glycoprotein (AGP) was performed with Roche Cobas Integra 400 Plus analyser (Roche Diagnostics GmbH, German). Hemoglobin levels <12.0 and <8.0 g/dL were used to characterise anaemia and severe anaemia, respectively. Serum C-reactive protein (CRP) and Alpha-1-acid glycoprotein (AGP) values of CRP > 5.0 mg/L and AGP > 1.0 g/L respectively were characterized as high inflammatory marks [18].

Data analysis

The data were analysed by using SPSS version 25. The ~~poor~~ dietary intake as dependent variable was assessed as a categorical variable splitting at the median i.e. 0 = good dietary intake ~~and diet quality~~ and, 1= poor dietary intake ~~and diet quality~~.

An asset-based approach to measuring household socio-economic status is considered an alternative to income and consumption expenditure in low-income countries. Principal Components Analysis (PCA) is a method for determining wealth indices [19]. In this study, household wealth index was assessed as (1) “available and in working condition” or (0) “not available and/or not in working condition” of durable assets, housing characteristics and access to basic services. For constructing a wealth index among pregnant women in Mbeya, the first principal component was used to categorise households into two approximate group’s i.e. lowest

and highest group. The strengths of the associations between the dependent and independent variables in bivariate analysis were tested using the Pearson chi-square tests because all variables were categorical. Independent variables that were significant at arbitrary levels in the bivariate analysis were selected for multivariate analysis. We based this on the Wald test with a P-value cut-off of 0.7. In multivariate analysis, Log binomial regression method were used first for adjusting confounders and second to identify independent predictors of ~~poor~~ dietary intake ~~and diet quality~~ among the study population, ~~. All tests were two-tailed~~ and the significance level was set at 5%.

Ethics Statement

The survey was approved by the Tanzania Ethics Committees i.e. the National Institute for Medical Research with the reference number SZEC-24239/R.A/V.1/151. Date of issue 12th August 2022. All eligible subjects were informed of the purpose and nature of the survey and those who agreed to participate were asked to sign a written informed consent form. Moreover, a written informed consent was ~~also~~ obtained from the parent/guardian of each participant under 18 years of age. All procedures followed were per the ethical standards of the Helsinki Declaration of 1975 including the confidentiality and, authors had no access to information that could identify individual participants during or after data collection.

Results

Reliability

The internal consistency reliability scales were examined using Cronbach's alpha. Test-retest reliability analysis was performed using kappa statistics and Intra class correlation coefficients (ICC). The agreement between the interviewers and the gold standard on the dietary intake ~~and~~

diet quality assessments on the English and Kiswahili versions were Cohen's kappa of 0.62 and 0.67 respectively. During the field, duplicate interviews were performed randomly with 20 pregnant women. Test-retest reliability of reports on the dietary intake and diet quality assessment using a Kiswahili version in terms of ICC was 0.72 (95% CI 0.64 – 0.78). Thus, acceptable levels of intra-interviews agreement (kappa >0.60) were obtained [20].

The characteristics of the study population

Study participants had a mean age of 25.49 ± 6.37 years. T**able 1** depicts the characteristics of the study population. More than half of the participants were 15-24 years old, about seventy-two percent had completed at least primary education and, 84.3% were self-employed. Five percent (n=21) of pregnant women had Mid Upper Arm Circumference (MUAC) of above 33cm and falls in the category of overweight and obesity. Nine percent (n=38) of pregnant women had serum C- reactive protein (CRP) above 5mg/L and, 19% (n=80) had Alpha-1-acid glycoprotein (AGP) above 1 g/L. For the construction of wealth index: One-third of the participants lived in houses with electricity; 72.0% had access to improved sources of drinking water and 65.0% were not sharing toilet facilities. The pit latrine without washable was the most common type of toilet 31.6 % (n=133). About half (51.5%) used cement as the material of the dwelling floor and 7.8% used thatch/palm leaf as material for the roof. Furthermore, less than two percent (n=6) and 2.4% (n=10) of the participants owned a motor vehicle and a set of television respectively (not in Table 1). Following the PCA analysis, 140 pregnant women (33.4%) fell under the category of lower socio-economic status as shown in Table 1

Dietary intake and diet quality

Among 420 pregnant women who participated in the study, two hundred forty (57.2%) fell into the group of poor dietary intake. The median PDQS was 16 (the 25th and 75th percentiles were

14.0 and 18.0, respectively). **Table 2** shows the patterns and distribution of dietary intake ~~and diet quality~~ among pregnant women according to PDQS on healthy and unhealthy food shows that 57.2 of the study participants consumed more than four servings of edible vegetable liquid oil per week out of the 14 healthy foods assessed. Furthermore, the healthy foods that were less consumed per week were cruciferous vegetables (93.4%), whole citrus fruits (92.9%), and poultry (92.4%)~~. About less than one-third of study participants (n=123) reported consuming more than four times dark green leafy green vegetables, about fifteen percent reported consuming other vegetables, and 57.2% edible vegetable liquid oil per week. Slightly similar trends for other healthy foods, including cruciferous vegetables, dark orange vegetables and fruits, whole citrus fruits, other whole fruits, legumes, nuts and seeds, poultry, fish, whole grains, white roots and tubers and low-fat dairy were also consumed infrequently by pregnant women~~. However, refined grains and baked goods was high (48.0%) high serving consumed per week out of the six unhealthy foods assessed. ~~and sugar-sweetened beverages were the most commonly consumed unhealthy foods~~.

Bivariate analysis

Table 3 depicts the bivariate analysis; poor dietary intake ~~and diet quality~~ were significantly associated with the marital status of pregnant women, and those who received Fansidar tablets during pregnancy (p>0.05). However, the age group of pregnant women, their educational level, occupation status, household wealth index, received iron and folic acid supplements, Serum C-reactive protein (CRP) and Alpha-1-acid glycoprotein (AGP) were not significantly associated with poor dietary intake and diet quality among pregnant women (p>0.05).

Multivariate analysis

Table 4 presented all variables which were significant at arbitrary levels in the ~~(p>0.7) at~~ bivariate analysis and hence qualify to be included in the multivariate analysis. Using a Log binomial regression method, the study found out that poor dietary intake ~~and diet quality~~ were less likely among cohabiting pregnant women [Adjusted RR 0.22 (95% CI 0.09- 0.50)] and; those who reported taking Fansidar (Sulfadoxine and Pyrimethamine, SP) tablets during pregnancy in Mbeya region [Adjusted RR 0.55 (95% CI 0.31- 0.96)]. Further, the study found that poor dietary intake ~~and diet quality~~ were more likely among pregnant women who were classified as overweight and obese by the MUAC [Adjusted RR 3.49 (95% CI 1.10- 11.06)] and; slightly significant among pregnant women of age group 25-29 years old [Adjusted RR 2.05 (95% CI 0.98- 4.29)]. Poor dietary intake ~~and diet quality were~~ was not associated with higher concentrations of inflammatory factors i.e. CRP and AGP ~~(p>0.05)~~.

Discussion

This study contributes to our understanding on socio-demographic drivers for poor dietary intake among pregnant women legally married or cohabiting in Tanzania. Worldwide, poor dietary intake has ~~seriously detrimental~~negative consequences for pregnancy and born children ~~both in developed and developing countries~~ [1, 21]. This study found that dietary intake among pregnant women in the rural settings of Tanzania was largely characterised by low intakes of fruits~~, grains~~ and vegetables. The findings are very similar to those found in Ethiopia [21], Iran [30], and India [31]. However, a study from the urban settings of Tanzania reported a high consumption of vegetables among ~~HIV-negative~~ pregnant women [22]. This could be explained by the difference in research methodology especially the inclusion and exclusion criteria of the study participants, and also the difference in dietary assessment tools used between the studies. Evidence shows that, the nutrients in fruits and vegetables such as fibers, vitamins, minerals, and phytochemicals

play a key role in human health and well-being [23, 25, 26, 27]. Tanzania has recently developed its national food based dietary guidelines and, it has not yet been fully operationalized. Though other countries like UK and USA their guidelines recommended a plant-based diet, rich in fruits, vegetables, whole grains, and legumes to lower the risk of heart disease, type 2 diabetes, obesity, and other health conditions [24].

In this study, we highlight the factors that affect the dietary intake of pregnant women in the rural settings of Tanzania. Our findings affirmed previous researches on the relationship between cohabitation and dietary intake among pregnant women [29]. Pregnant women who engaged in cohabiting relationships seemed to have better education and financial position and, thus reflected on their decision power over their wealth [29]. From the anecdotal evidence, it is known that cohabiting relationships are a common practice in the study area. According to the study of Dinour and colleagues in 2012, they found that marital status is one of strong socio-demographic factors that greatly influence health-related behaviours and outcomes [28]. However, studies emanating from sub-Saharan African countries merge cohabitation and marriage into one category of marital status and, this is because pregnant women are reluctant to report the status of cohabitation because of stigma [32]. This study managed to assess separate the marriage and cohabitation statuses because there are often different outcomes for the health and well-being of pregnant women and their children in different settings. Further prospective cohort research is needed to investigate the social aspects that link marital transition and dietary intake outcomes among pregnant women in sub-Saharan African countries.

Parallel with globalisation, pronounced changes in the human behaviour and lifestyle such as decreased consumption of fruits and vegetables and increased consumption of unhealthy foods [32], have resulted in escalating rates of overweight and obesity among pregnant women. The

trends of overweight and obesity among pregnant women in Tanzania has changed from being considered as a mild disorder to the major causes of morbidity and mortality associated with non-communicable diseases [7]. According to MUAC measures, this study found that only twenty one pregnant women had MUAC measures above 33cm [42]. Further, our study revealed that out of 420 pregnant women, only 12.6% and 29.3% consumed at least four servings of fruits and vegetables per week respectively. Similar findings were documented in the previous studies from low-income countries, where overweight and obese pregnant women consumed less vegetable and fruits [21, 34]. In multivariate analysis, our data revealed that pregnant women who were overweight or obese ~~(MUAC measures above 33 cm)~~ were significantly associated with poor dietary intake. Pregnant women with overweight and obesity need nutrition counseling that are supported by scientific evidence and that can be easily understood and translated into everyday life to improve maternal and birth outcomes. Finding from a large, long-term randomised controlled trials provide convincing evidence that changes made in physical activity levels and dietary habits are effective in delaying, and possibly preventing, progression from overweight and obesity to non-communicable diseases [39]. Future prospective cohort research is needed to investigate the link between obese and dietary patterns among pregnant women.

~~The World Health Organisation (WHO) recommended Intermittent Presumptive Treatment (IPT) to pregnant women to prevent the adverse effects of malaria during pregnant.~~ This study also highlighted important finding, the protective effect to poor dietary intake among pregnant women who reported taking SP tablets~~ (IPT) during their pregnancy~~. In Tanzania, SP tablets are ~~mandatory~~ offered ~~-~~to all pregnant women attending antenatal clinics between 16 and 24 weeks ~~for the first IPT~~ and, between 28 and 32 weeks ~~for the second IPT~~ [40]. The linkage between taking SP tablets and poor dietary intake protection could not established because malaria and

nutrition interventions are well integrated into antenatal care in Tanzania, and both impart pregnant women with essential knowledge~~The increase in percentage of pregnant women who use SP for IPT and, protected from poor dietary intake, possibly due to the fact that majority of the study population made early antenatal booking, availability of integrated nutrition and malaria intervention and, a result of continued national advocacy and sensitisation carried out in the country by various stakeholders~~ [41]. Further studies are needed to broaden the understanding on this relationship and, help researchers in sub-Saharan African countries to develop tailored interventions to improve maternal and birth outcomes.

Strengths and limitations

There are several strengths of this study. First, the study was able to use reliable survey data and blood samples from a large population sample and measured diet during the pregnancy using both the Prime Diet Quality Score tool and 24-hour dietary recall. Second, this study provides important information on factors associated with ~~poor~~ dietary intake among pregnant women of gestation period less than 28 weeks, which is important for fetal development, given the rapid cell growth, and development of immune cells and organs in the first trimester [35, 36]. Third, the present study is that it links social and biological data with dietary data and allows analysis of dietary intake. However, there were several limitations of the study. First, we inevitably have some level of measurement error in both dietary and social and biological data, as both were based on self-report. This source of error is, however, expected to be largely random, producing valid estimates for the study population. Second, we derived PDQS scores from 24-hour recalls, and there were limited precedents in published literature for converting these scores to equivalent scores for the food frequency questionnaire (FFQ). The validity of using the PDQS score assessed using 24-hour recall is an area of active research. Notably, the 24-hour recall method is

used widely in developing countries and our findings provide support for the use of this metric for deriving PDQS in these settings. Our findings may not be generalisable to populations where dietary patterns and determinants outcomes differ from rural Tanzania. Associations may be stronger in populations with more prevalent micronutrients and other deficiencies in pregnant women.

Conclusions

The results of this study affirm that cohabitation and obesity affect dietary intake among pregnant women differently compared to marriage in rural settings of Tanzania. Further, the findings suggest that public health action is needed to promote the consumption of fruits and vegetables among pregnant women in Mbeya. We recommend prospective cohort research to investigate the social aspects that link poor dietary intake outcomes for developing a tailored gestational intervention to improve maternal and birth outcomes in sub-Saharan African countries.

Supporting information

Strobe statement (strobe_statement_dietary.docx)

Copy of ethical clearance (Ethical clearance.pdf)

List of Abbreviation

MUAC: mean upper arm circumference

PDQS: Prime diet quality score

RCH: Reproductive and Child Health

SDG: Sustainable Development Goals

TNNS: National Nutrition Survey

Declarations:

Institutional Review Board Statement

The protocol and data collection procedures for this survey were approved by the Tanzania Ethics Committees i.e. the National Institute for Medical Research with the reference number SZEC-24239/R.A/V.1/151.

Informed Consent Statement

All participants were asked to provide written consent after being read a document emphasizing the voluntary nature of the survey. The risks and benefits of the study were clearly explained to the participants before signing the consent. Also participants were informed of their rights to refuse to participate in the study or withdraw from it at any time, without consequences. All procedures followed were per the ethical standards of the Helsinki Declaration of 1975 including the confidentiality.

Data Availability Statement

All datasets underlying this study are freely available at the public repository https://osf.io/7ysb9/.

Competing interests
The authors declare that they have no competing interests.

Funding
This research received no specific financial support

Authors' contributions
Conceptualization, EK, HM, AH, KA, NN, TL, GM, RN, RM, AS, RN, PC, GHL and RMM; project administration and resources, RMM and GHL; formal analysis and writing—original draft, EK, HM, AH, KA, NN, TL, GM, RN, RM, AS and RN; writing—review and editing, EK, HM, AH, KA, NN, TL, GM, RN, RM, AS, RN, PC, GHL and RMM. All authors have read and agreed to the published version of the manuscript.

Reference

1. Latal-Hajnal B, von Siebenthal K, Kovari H, Bucher HU, Largo RH. Postnatal growth in VLBW infants: significant association with neurodevelopmental outcome. The Journal of pediatrics. 2003;143:163-70.
2. Pelletier JE, Laska MN, MacLehose R, Nelson TF, Nanney MS. Evidence-based policies on school nutrition and physical education: Associations with state-level collaboration, obesity, and socio-economic indicators. Preventive medicine. 2017;99:87-93.
3. Ahmad S, Moorthy MV, Demler OV, Hu FB, Ridker PM, Chasman DI, et al. Assessment of Risk Factors and Biomarkers Associated With Risk of Cardiovascular Disease Among Women Consuming a Mediterranean Diet. JAMA Network Open. 2018;1:e185708.
4. Lopez-Garcia E, Rodriguez-Artalejo F, Li TY, Fung TT, Li S, Willett WC, et al. The Mediterranean-style dietary pattern and mortality among men and women with cardiovascular disease. AJCN. 2013;99:172-80.
5. Afshin A, Sur PJ, Fay KA, Cornaby L, Ferrara G, Salama JS, et al. Health effects of dietary risks in 195 countries, 1990–2017: a systematic analysis for the Global Burden of Disease Study 2017. The lancet. 2019;393:1958-72.
6. Venter IM, Winterbach A. Dietary fat knowledge and intake of mid-adolescents attending public schools in the Bellville/Durbanville area of the city of Cape Town. South African Journal of Clinical Nutrition. 2010;23.
7. Tanzania National Nutrition Survey, TNNS, 2014 final report. Tanzania Food and Nutrition Centre (TFNC), Ministry of Health and Social Welfare. 2015, Dar es Salaam. 2018.
8. Gharib N, Rasheed P. Energy and macronutrient intake and dietary pattern among school children in Bahrain: A cross-sectional study. Nutr J. 2011;10:1-12.
9. Bhanbhro S, Kamal T, Diyo RW, Lipoeto NI, Soltani H. Factors affecting maternal nutrition and health: A qualitative study in a matrilineal community in Indonesia. Plos one. 2020;15:e0234545.
10. Popkin, B.M., Synthesis and implications: C hina's nutrition transition in the context of changes across other low- and middle- income countries. Obesity reviews. 2014;15:60-67.
11. Mayen AL, Marques-Vidal P, Paccaud F, Bovet P, Stringhini S. Socioeconomic determinants of dietary patterns in low-and middle-income countries: a systematic review. The American journal of clinical nutrition. 2014;100:1520-31.
12. Azizan NA, Thangiah N, Su TT, Majid HA. Does a low-income urban population practise healthy dietary habits?. International health. 2018;10:108-15.
13. Gicevic S, Gaskins AJ, Fung TT, Rosner B, Sabanovic E, Milesevic J, et al. Demographic and socio-economic predictors of diet quality among adults in Bosnia and Herzegovina. Public health nutrition. 2019;22:3107-17.
14. Coates JC, Colaiezzi BA, Bell W, Charrondiere UR, Leclercq C. Overcoming dietary assessment challenges in low-income countries: technological solutions proposed by the International Dietary Data Expansion (INDDEX) Project. Nutrients. 2017;9:289.
15. Tanzania National Bureau of Statistics, NBS 2013. Population and housing census 2012.

16. Lwanga SK, Lemeshow S, World Health Organization. Sample size determination in health studies: a practical manual. World Health Organization; 1991. (Accessed on January 2022. URL: https://apps.who.int/iris/handle/10665/40062).
17. Fung TT, Isanaka S, Hu FB, Willett WC. International food group–based diet quality and risk of coronary heart disease in men and women. The American journal of clinical nutrition. 2018;107:120-9.
18. World Health Organization. (2020). WHO guideline on use of ferritin concentrations to assess iron status in individuals and populations. World Health Organization. URL: https://apps.who.int/iris/handle/10665/331505 (Accessed June 2023)
19. Vyas S, Kumaranayake L: Constructing socio-economic status indices: how to use principal components analysis. Health Policy Plan. 2006, 21: 459-468.
20. Landis JR, Koch GG: An application of hierarchical kappa-type statistics in the assessment of majority agreement among multiple observers. Biometrics 1977, 33:363–374.
21. Zerfu TA, Pinto E, Baye K. Consumption of dairy, fruits and dark green leafy vegetables is associated with lower risk of adverse pregnancy outcomes (APO): a prospective cohort study in rural Ethiopia. Nutr Diabetes. 2018;8:52.
22. Madzorera I, Isanaka S, Wang M, Msamanga GI, Urassa W, Hertzmark E, et al. Maternal dietary diversity and dietary quality scores in relation to adverse birth outcomes in Tanzanian women. The American journal of clinical nutrition. 2020;112:695-706.
23. Slavin JL, Lloyd B. Health benefits of fruits and vegetables. Adv Nutr. 2012;3:506-16.
24. Thompson HJ. The dietary guidelines for Americans (2020–2025): pulses, dietary fiber, and chronic disease risk—a call for clarity and action. Nutrients. 2021;13:4034.
25. King, J.C., Physiology of pregnancy and nutrient metabolism. The American journal of clinical nutrition. 2000;**71**:1218S-1225S.
26. Kind KL, Moore VM, Davies MJ. Diet around conception and during pregnancy–effects on fetal and neonatal outcomes. Reproductive biomedicine online. 2006;12:532-41.
27. Imdad A, Bhutta ZA. Nutritional management of the low birth weight/preterm infant in community settings: a perspective from the developing world. The Journal of pediatrics. 2013;162:S107-14.
28. Dinour L, Leung MM, Tripicchio G, Khan S and MC Yeh The Association between Marital Transitions, Body Mass Index, and Weight: A Review of the Literature. J. Obes. 2012; 2012.
29. Dallmann D, Marquis GS, Colecraft EK, Dodoo ND. Marital transition is associated with food insecurity, low dietary diversity, and overweight in a female population in rural Ghana. African Journal of Food, Agriculture, Nutrition and Development. 2023;23:22149-71.
30. Akbari Z, Mansourian M, Kelishadi R. Relationship of the intake of different food groups by pregnant mothers with the birth weight and gestational age: Need for public and individual educational programs. Journal of education and health promotion. 2015;4.
31. Rao S, Yajnik CS, Kanade A, Fall CH, Margetts BM, Jackson AA, et al. Intake of micronutrient-rich foods in rural Indian mothers is associated with the size of their babies at birth: Pune Maternal Nutrition Study. The Journal of nutrition. 2001;131:1217-24.
32. Marston M, Slaymaker E, Cremin I, Floyd S, McGrath N, Kasamba I, et al. Trends in marriage and time spent single in sub-Saharan Africa: a comparative analysis of six

population-based cohort studies and nine Demographic and Health Surveys. Sexually transmitted infections. 2009;85:i64-71.

33. Stuebe AM, Oken E, Gillman MW. Associations of diet and physical activity during pregnancy with risk for excessive gestational weight gain. American journal of obstetrics and gynecology. 2009;201:58-e1.
34. George GC, Milani TJ, Hanss-Nuss H, Freeland-Graves JH. Compliance with dietary guidelines and relationship to psychosocial factors in low-income women in late postpartum. Journal of the American Dietetic Association. 2005 Jun 1;105(6):916-26.
35. Palmer AC. Nutritionally mediated programming of the developing immune system. Advances in nutrition. 2011;2:377-95.
36. Gruszfeld D, Socha P. Early nutrition and health: short-and long-term outcomes. InEvidence-Based Research in Pediatric Nutrition. Karger Publishers.2013;108: 32-39.
37. World Health Organisation, Food Based Dietary Guidelines in the WHO European Region. 2003. URL: http://www.euro.who.int (Accessed June 2023)
38. Culliford AE, Bradbury J, Medici EB. Improving Communication of the UK Sustainable Healthy Dietary Guidelines the Eatwell Guide: A Rapid Review. Sustainability. 2023;15:6149.
39. Harris SB, Petrella RJ and Leadbetter W. Lifestyle interventions for type II diabetes relevance for clinical practice. Canadian Family Physician. 2003;49:1618-1625.
40. Falade CO, Yusuf BO, Fadero FF, Mokuolu O, Hamer DH, Salako L. Intermittent preventive treatment with sulphadoxine-pyrimethamine is effective in preventing maternal and placental malaria in Ibadan, south-western Nigeria. Malaria J. 2007;6:1–8.
41. Mubyazi GM, Bygbjerg IC, Magnussen P, Olsen O, Byskov J, Hansen KS, et al. Prospects, achievements, challenges and opportunities for scaling-up malaria chemoprevention in pregnancy in Tanzania: the perspective of national level officers. Malaria J. 2008;135:1–6.
42. Okereke CE, Anyaehie UB, Dim CC, Iyare EE, Nwagha UI. Evaluation of some anthropometric indices for the diagnosis of obesity in pregnancy in Nigeria: a cross-sectional study. African health sciences. 2013;13:1034-40.
43. Strong C. Minimal risk in research involving pregnant women and fetuses. J Law Med Ethics. 2011;39:529-38.
44. Logan D, McEvoy CT, McKenna G, Kee F, Linden G, Woodside JV. Association between oral health status and future dietary intake and diet quality in older men: The PRIME study. Journal of dentistry. 2020;92:103265.
~~41.~~45. Gicevic S, Gaskins AJ, Fung TT, Rosner B, Sabanovic E, Milesevic J, et al. Demographic and socio-economic predictors of diet quality among adults in Bosnia and Herzegovina. Public health nutrition. 2019;22:3107-17.

LIST OF TABLES

Table 1: Frequency distribution of the socio-demographic and economic characteristics of pregnant women in Mbeya (n=420)

Variable	Categories	Frequency (n)	Percentage (%)
Age group (Years)	15-19	82	19.5
	20-24	133	31.6
	25-29	99	23.6
	35+	106	25.2
Education	No education	34	8.1
	Primary	301	71.7
	Secondary and above	85	20.2
Marital status	Married	238	56.5
	Cohabit	133	31.6
	Single/ Divorced	49	11.6
Number of pregnancies	Primigravida	104	24.7
	Multigravida	316	75.1
Trimester of pregnancy	First trimester (<12 weeks)	109	26.0
	Second trimester (12-26 weeks)	311	74.0
Received of iron and folic acid supplements	No	155	36.8
	Yes	265	62.9
Received Fansidar (SP) during pregnancy	No	204	48.5
	Yes	216	51.3
Occupation status	Formal employment	15	3.6
	Self employed	355	84.3
	Not employed	51	12.1
Household wealth Index	Higher socio-economic	140	33.4
	Middle socio-economic	139	33.2
	lower socio-economic	140	33.4
Mid Upper Arm Circumference (MUAC)	Thin (<23cm)	16	3.8
	Normal (between 23 and	383	91.2

	33cm)		
	Overweight or obesity (above 33 cm)	21	5.0
Malaria infection	No	402	95.7
	Yes	18	4.3
Serum C- reactive protein (CRP)	CRP≤5mg/L	383	91.0
	CRP>5mg/L	38	9.0
Alpha-1-acid glycoprotein (AGP)	AGP<=1 g/L	341	81.0
	AGP>1 g/L	80	19.0

Table 2: The patterns and distribution of food groups consumption among pregnant women according to PDQS score (n=420)

**Healthy foods**			
Serving per week	***0–1 serving/week n (%)***	***2–3 servings/week n (%)***	***≥4 servings/week n (%)***
Dark leafy green vegetables	142(33.7)	156 (37.1)	123 (29.2)
Cruciferous vegetables	393(93.4)	20(4.8)	8(1.9)
Dark orange vegetables and fruits	263(62.5)	105(24.9)	53(12.6)
Other vegetables	266(63.2)	93 (22.1)	62(14.7)
Whole citrus fruits	391 (92.9)	25 (5.9)	5 (1.2)
Other whole fruits	308(73.2)	78(18.5)	35(8.3)
Legumes	259 (61.5)	104 (24.7)	58(13.9)
Nuts and seeds	275(65.3)	107(25.4)	39(9.3)
Poultry	389(92.4)	21(5.0)	11(2.6)
Fish	277(65.8)	98(23.3)	46(10.9)
Whole grains	324(77.0)	68(16.1)	29(6.9)
Vegetable liquid oils	46(10.9)	134(31.8)	241(57.2)
White roots and tubers	189(44.9)	161(38.2)	71(16.9)
Low fat diary	349(82.9)	56(13.3)	16(3.8)
**Unhealthy foods**			
Serving per week	***0–1 serving/week n (%)***	***2–3 servings/week n (%)***	***≥4 servings/week n (%)***
Red meats	311(73.9)	85(20.2)	25(5.9)
Sweets and ice cream	349(82.9)	58 (13.8)	14 (3.3)
Fried foods obtained away from Home	348(82.7)	65(15.4)	8(1.9)
Processed meat	412(97.9)	8(1.9)	1(0.2)
Refined grains and baked goods	74(17.6)	145(34.4)	202 (48.0)
Sugar sweetened beverages	249(59.1)	146(34.7)	26(6.2)

Table 3: Bivariate analysis on the factors associated with poor dietary quality among pregnant women in Mbeya (n=420): **Chi square test**

Variable	Categories	Good dietary quality	Poor dietary quality	P-value
		% (n)	*% (n)*	
Age group (Years)	15-19	16.8 (30)	21.6 (52)	0.129
	20-24	30.7 (55)	32.4 (78)	
	25-29	29.1 (52)	19.5 (47)	
	35+	23.5 (42)	26.6 (64)	
Education	No education	8.3 (15)	7.9 (19)	0.696
	Primary	70.6 (127)	72.6 (175)	
	Secondary and above	21.1 (38)	19.5 (47)	
Marital status	Married	70.0 (126)	46.5 (112)	0.000
	Cohabit	17.8 (32)	42.3 (102)	
	Single/ Divorced	12.2 (22)	11.2 (27)	
Trimester of pregnancy	First trimester (<12 weeks)	27.8 (50)	24.9 (60)	0.506
	Second trimester (12-26 weeks)	72,2 (130)	75.1 (181)	
Received of iron and folic acid supplements	No	38.5 (69)	35.7 (86)	0.548
	Yes	61.5 (110)	64.3(155)	
Received Fansidar during pregnancy	No	43.6 (78)	52.3 (126)	0.077
	Yes	56.4 (101)	47.7 (115)	
Occupation status	Formal employment	5.6 (10)	2.1 (5)	0.156
	Self employed	83.3 (150)	85.5 (206)	
	Not employed	11.1 (20)	12.4 (30)	
Household wealth Index	Higher socio-economic	33.0 (59)	34.0 (82)	0.365
	Middle socio-economic	30.2 (54)	35.3 (85)	
	lower socio-economic	36.9 (66)	30.7 (74)	

Mid Upper Arm Circumference (MUAC)	Thin (<23cm)	3.9 (7)	3.7 (9)	0. 079
	Normal (between 23 and 33cm)	91.6 (164)	90.9 (219)	
	Overweight or obesity (above 33 cm)	4.5 (8)	5.4 (13)	
Malaria infection	No	96.7 (174)	95.0 (229)	0.409
	Yes	3.3 (6)	5.0 (12)	
Serum C-reactive protein (CRP)	CRP≤5mg/L	89.4 (161)	92.1 (222)	0.344
	CRP >5mg/L	50.0 (19)	7.9 (19)	
Alpha-1-acid glycoprotein (AGP)	AGP<=1 g/L	80.6 (145)	81.3 (196)	0.542
	AGP>1 g/L	19.4 (35)	18.7 (45)	

Table 4: Multivariate Log binomial regression methods were used to assess factors associated with poor dietary intake among pregnant women in Mbeya (n=420).

			**95% confidence interval for Adj. RR**	
**Variable**	**Category**	**Adjusted RR**	**Lower bound**	**Upper bound**
**Age group**	15-19	1.22	0.51	2.89
	20-24	1.31	0.66	2.59
	25-29	**2.05**	**0.98**	**4.29**
	35+	1	1	1
Marital status	Married	1.35	0.60	3.04
	Cohabit	**0.22**	**0.09**	**0.50**
	Single/divorced	1	1	1
Occupational status	Formal employment	4.29	0.83	22.17
	Self employed	1.02	0.45	2.29
	Not employed	1	1	1
Education level	No formal	0.73	0.21	2.56

	Primary	0.95	0.47	1.91
	Secondary and above	1	1	1
Household wealth index	Higher socio-economic	1.40	0.77	2.56
	Middle socio-economic	0.70	0.36	1.35
	lower socio-economic	1	1	1
Mid Upper Arm Circumference (MUAC)	Thin (<23cm)	3.02	0.52	17.50
	Overweight or obesity (above 33 cm)	**3.49**	**1.10**	**11.06**
	Normal (between 23 and 33cm)	1	1	1
Taken Fansidar during this pregnancy	Yes	**0.55**	**0.31**	**0.96**
	No	1	1	1
Malaria status	Yes	1.89	0.54	6.62
	No	1	1	1
Received of iron and folic acid supplements	Yes	1.09	0.62	1.90
	No	1	1	1
Alpha-1-acid glycoprotein (AGP)	AGP<=1 g/L	0.94	0.48	1.81
	AGP>1 g/L	1	1	1
Serum C- reactive protein (CRP)	CRP≤5mg/L	0.60	0.24	1.50
	CRP>5mg/L	1	1	1
Number of pregnancy	Primigravida	1.09	0.54	2.22
	Multigravida	1	1	1
Trimester of pregnancy	First trimester (<12 weeks)	1.14	0.64	2.03
	Second trimester (12-26 weeks)	1	1	1

The reference group is last category

Dear Dickson Abanimi Amugsi, PhD

Thank you very much for giving us the opportunity to submit a revised draft of our manuscript titled “Dietary intake and associated risk factors among pregnant women in Mbeya, Tanzania” We appreciate the time and effort that you and reviewers have dedicated to providing valuable feedback on our manuscript.
Here is a point-by-point response to the editor and reviewers’ comments and concerns.

Journal Requirements:
Comment 1:
We noticed you have some minor occurrence of overlapping text with the following previous publication(s), which needs to be addressed:

-https://link.springer.com/content/pdf/10.1186/s13104-016-2122-3.pdf
-https://journals.plos.org/globalpublichealth/article?id=10.1371%2Fjournal.pgph.0001828
-https://pureadmin.qub.ac.uk/ws/files/195797445/Association_between_oral_health_status_and_future_dietary_intake_and_diet_quality_in_older_men_the_PRIME_study.pdf

In your revision ensure you cite all your sources (including your own works), and quote or rephrase any duplicated text outside the methods section. Further consideration is dependent on these concerns being addressed."""
Response: Thank you very much for pointing out this. We agree with your comment and made citations as suggested.

Review Comments to the Author
Reviewer #1
Comment 1: *line 123 &126/7- Provide a rationale and justification for exclusion criteria e.g why cut off at 28 weeks gestational age when the title of the article includes ‘pregnant women’ and why exclude pregnant woman taking medication and define the criteria used to define medication as shall be highlighted later in the review.*
Response: Thank you very much and we agree with your comment. We have indicated the rationale and justification for exclusion of pregnant women of gestation week more than 28 weeks (line number 125-126) and, describing kinds of medication excluded in this study (line number 129).

Comment 2: *Line 144- provide a rationale for use of a screening tool (PDQS) that has not been tested in the population group of interest which is pregnant women in low to middle income countries (provide evidence if the case is otherwise). This is because even though the authors acknowledge the limitations of their choice of screening tool (PDQS in this case), the identification of the ‘at risk’ pregnant women is highly dependent on the validity, sensitivity and specificity of this screening tool and conclusions made should be translated and synthesized after acknowledging the strengths and weaknesses of the screening tool and why it was used despite the identified/known strengths/weakness. This critical analysis of the tool can be analyzed in a thorough and similar manner to the method of its translation to KiSwahili which appear to have been executed in an excellent manner.*
Response: Thank you very much and we agree with your comment. We have rephrased the

sentence and insert references that provide a rationale for the use of PDQS (line numbers 148-151).

Comment 3: *line 163- The recall bias associated with a 24 hr recall has be acknowledged and a 30 day recall might have brought a different comparable outcome that could also be acknowledged in an obvious attempt to include a wider dietary assessment but this is speculation from a reviewer who shares the opinion that acknowledging such gaps might strengthen the recommendations for future research practice.*

Response:

Thank you very much and we agree with your comment. We have inserted the sentence about recall bias associated with 30-day recall (line number 168- 169).

Comment 4: *Line 267- Elaborate on the inclusion of pregnant women taking Fansidar (line 267,278, 336), iron and folic acid (line270) (chemicals that the reviewer considers to be medication) when the exclusion criteria excluded women taking medication.*

Response: Thank you very much for pointing this out and, we have added information to clarify the inclusion of pregnant women who are taking Fansidar (line number 129-130).

Comment 5: *285- The discussion appears to underreport the relationship between the socioeconomic environment or disease states like anemia, hypoalbuminemia etc to dietary intake even though such indices were reported to have been captured in the method and results. As a reader i remain unquenched as to whether the relationship was not reported because the relationship was insignificant to report or it was simple neglect..*

Response: Thank you very much for pointing this out. After controlling for the cofounders and effect modifiers, we could not establish a significant relationship between the socioeconomic, environment or disease states with dietary intake.

<u>Reviewer #2</u>

Comment 1: *We do not assess poor dietary intake, rather dietary intake to ascertain if its poor or not*

Response: Thank you very much and we agree with your comment. We have made that correction throughout the manuscript.

Comment 2: *in the methods: concern on authors assessing {"poor diet") poor diet is an outcome of dietary assessment. You can only determine of the respondents consumed poor diet or good diet after the assessment. It is a product of assessment. I am not sure there is a tool designed to assess poor diet. We have tools to assess dietary intake. Analysis will confirm if the diet was poor or not. If the authors have to focus on poor diets, then they need to state of there are other findings that poor diets in the study population that has led to this study to assess the factors contributing to poor intake. However, if there is no primary data on this, then the focus should be to assess dietary intake.*

Response: We highly appreciate the insightful comments and, we have made the correction as suggested.

Comment 3: *There is need to review the statements in the whole manuscript, e.g. the statement "A cross-sectional study design was conducted in Mbeya" should delete the word design*

Response: Thank you very much for pointing this out and, we have deleted the word ‘design’ as suggested.

Comment 4: *The refined grains are portrayed as a good thing while in essence the focus of assessment should be the proportion of mothers consuming unrefined grains.*
Response: Thank you very much and we agree with your comments. We have rephrased the paragraph accordingly (line number 271-282).

Comment 5: *Poor dietary intake has seriously detrimental consequences for pregnancy and born children both in developed and developing countries (this statement has been overused, in the abstract, introduction and discussions)*
Response: Thank you very much and we agreed with the comments and made corrections as suggested both in the abstract, introduction, and discussions.

Comment 6: *In the discussion, the comparison between pregnant women and women with HIV may not be the ideal. Persons with HIV tend to receive a lot of nutrition education to improve their nutrition status given the effects of poor nutrition on HIV outcomes.*
Response: Thank you very much for pointing this out. For clarity, we deleted the words ‘HIV-negative’ (line number 311).

Comment 7: *The findings and the title need to be aligned better; the results have had very specific association cohabiting, obesity, and malaria treatment. Maybe the title should consider capturing this. From the findings cohabiting did promote good dietary intake, it therefore does not qualify to be a factor of poor dietary intake.*
Response: Thank you very much and we agree with your comment. In the appropriate areas, the word ‘poor’ was deleted.

Comment 8: *The title suggests factors associated with poor dietary intake. Since the title has a bias on the poor intake, then emphasis on this should be discussed. It would be good to highlight possible factors that make cohabiting promote good dietary intake, this is not clear.*
Response: We agree with your comment and made changes as suggested.

Comment 9: *I also note only 5 % of the women were obese/overweight, but that’s also quite a small proportion and wondering if this is sufficient to draw the conclusion on effects of obesity on poor dietary intake. What is the link, a review of more literature for the discussions.*
Response: Thank you very much and we agreed with comments. We have re-written the sentences (line number 340-342 and 352-353).

Comment 10: *Fansider intake seems to have promoted dietary intake, however authors should discuss why this is so. How does malaria treatment improve dietary intake?*
Response: Thank you very much and we agreed with comments. We have re-written the paragraph for the clarity (line number 357-362).

Comment 11: *The only dietary items focused on in the results are vegetables and fruits and partly grains, why haven’t the authors mentioned the other foods? Were they not of interest to pregnant*

women especially proteins (animal sources) given the nutrient needs in pregnancy? Especially iron?
Response: Thank you very much for pointing this out. All dietary items findings were presented (line number 270-275) and discussed depend on their significance.

<u>Reviewer #3</u>
Comment 1: *Language editing is required. I started to correct some of the grammatical errors, but it became evident that there are many errors that will require language editing to fix these.*
Response: Thank you very much and, we highly appreciate the insightful and helpful comments on our manuscript. We agree with your comment and edited the language.

Comment 2: *Dietary intake and diet quality assessment: More detail on the exact process followed to collect dietary intake data is required. Please see manuscript provided for more feedback.*
Response: Thank you very much for pointing this out and we have re-checked and addressed small missing parts in the method section.

Comment 3: *I am also concerned about the relevance of using a tool developed for coronary heart disease risk when numerous other tools/instruments are available to determine dietary quality in pregnancy.*
Response: Thank you very much for pointing this out. The main reason behind selecting this tool is the fact that it has already been used among women of reproductive age in Tanzania (line number 149-151).

Comment 4: *Discuss anthropometric measurements under a separate heading. Also, please indicate which standardised techniques and/or references were used for measuring anthropometry.*
Response: We have added a sub-section of anthropometric measurements and also indicated citation (line number 193-199).

Comment 5: *Data analysis: Please indicate what cut-off level was used when reporting on P-values.*
Response: The cut-off level of statistical significance was P-value = 0.05. The arbitrary statistic level of 0.7 was only used for selecting variables for multivariate analysis.
Comment 6: *Ethics statement: Please indicate whether informed assent was obtained from minors.*
Response: Thank you very much for pointing this out. Informed consent was obtained from minors (pregnant women of age below 18 years).

Comment 7: *Dietary intake and diet quality: What was the mean/median PDQS score and how many women in the total sample were classified as having poor PDQS score?*
Response: We agreed with your comment. We have added a sentence for median PDQS score and, the total number of pregnant women classified as having poor PDQ score (line number 268-270).

Comment 8: *This entire section (lines 258 - 265) needs to be rephrased since the text does not clearly explain what is indicated in Table 2.*
Response: We agree with your comment and rephrased the entire section (line number 270-282).

Comment 9: *Sentence in lines 261 - 264 needs to be rephrased as it does not make sense currently.*
Response: We agree with your comment and rephrased the sentence.

Comment 10: *Lines 264 - 265: "Refined grains, baked goods and sugar-sweetened beverages were the most commonly consumed unhealthy foods" - According to the data presented in Table 2, 6.2% of women consumed four servings or more from the sugar sweetened beverages group per week? Thus it does not make sense how you make this statement?*
Response: Thank you very much and we agree with your comment and rephrased the sentence.

Comment 11: *Bivariate analysis:*
Adequately described. Please make sure of P-values, since the P-value for those who received Fansidar tables during pregnancy is indicated as 0.077 in the table, but in the text it is flagged as being significant? It will also be good to explain (either in the introduction or discussion) what these tablets are given for and how often.
Response: We have added more description on P-value cut-off used at the bivariate and multivariate analysis (line number 226-228).

Comment 12: *Multivariate analysis: Again, please verify what cut-off level/value was used when reporting on P-values. No P-values indicated in Table 4, thus the reference to P-value in line 284 does not make sense.*
Response: We agree with your comment and we have deleted the P-value indicated the line 284.

Comment 13: *Discussion: Needs language editing.*
Also consider differences on dietary assessment tools used ad possible reasons for differences between findings from the current study and that of others (line 294).
Response: We agree with your comments. We have edited the language throughout the manuscript and add the reasons for differences between findings from the current study and that of others.

Comment 14: *Lines 333-344: Regarding IPT needs to be discussed in context of the findings of the current study.*
Response: Thank you very much for pointing this out. We have rephrased the paragraph.

Sincerely,

Ray Masumo, PhD
