# Supplementary material for: Dietary intake and associated risk factors among pregnant women in Mbeya, Tanzania
Source: PLOS Glob Public Health. 2024 Jan 5;4(1):e0002529. doi: 10.1371/journal.pgph.0002529 (PMC10769095; doi:10.1371/journal.pgph.0002529)
Supplement: Attachment — Submitted filename: A rebuttal letter_dietary intake_03_10_2023.docx [file pgph.0002529.s003.docx]

Dear Dickson Abanimi Amugsi, PhD

Thank you very much for giving us the opportunity to submit a revised draft of our manuscript titled “Dietary intake and associated risk factors among pregnant women in Mbeya, Tanzania”
We appreciate the time and effort that you and reviewers have dedicated to providing valuable feedback on our manuscript.

Here is a point-by-point response to the editor and reviewers’ comments and concerns.

**Journal Requirements:**

**Comment 1:**

*We noticed you have some minor occurrence of overlapping text with the following previous publication(s), which needs to be addressed:*

*-https://link.springer.com/content/pdf/10.1186/s13104-016-2122-3.pdf
-https://journals.plos.org/globalpublichealth/article?id=10.1371%2Fjournal.pgph.0001828
-https://pureadmin.qub.ac.uk/ws/files/195797445/Association_between_oral_health_status_and_future_dietary_intake_and_diet_quality_in_older_men_the_PRIME_study.pdf*

*In your revision ensure you cite all your sources (including your own works), and quote or rephrase any duplicated text outside the methods section. Further consideration is dependent on these concerns being addressed."""*

**Response**: Thank you very much for pointing out this. We agree with your comment and made citations as suggested.

**Review Comments to the Author**

**Reviewer #1**

**Comment 1**: *line 123 &126/7- Provide a rationale and justification for exclusion criteria e.g why cut off at 28 weeks gestational age when the title of the article includes ‘pregnant women’ and why exclude pregnant woman taking medication and define the criteria used to define medication as shall be highlighted later in the review.*
**Response:** Thank you very much and we agree with your comment. We have indicated the rationale and justification for exclusion of pregnant women of gestation week more than 28 weeks (line number 125-126) and, describing kinds of medication excluded in this study (line number 129).

**Comment 2:** *Line 144- provide a rationale for use of a screening tool (PDQS) that has not been tested in the population group of interest which is pregnant women in low to middle income countries (provide evidence if the case is otherwise). This is because even though the authors acknowledge the limitations of their choice of screening tool (PDQS in this case), the identification of the ‘at risk’ pregnant women is highly dependent on the validity, sensitivity and specificity of this screening tool and conclusions made should be translated and synthesized after acknowledging the strengths and weaknesses of the screening tool and why it was used despite the identified/known strengths/weakness. This critical analysis of the tool can be analyzed in a thorough and similar manner to the method of its translation to KiSwahili which appear to have been executed in an excellent manner.*
**Response:** Thank you very much and we agree with your comment. We have rephrased the sentence and insert references that provide a rationale for the use of PDQS (line numbers 148-151).

**Comment 3**: *line 163- The recall bias associated with a 24 hr recall has be acknowledged and a 30 day recall might have brought a different comparable outcome that could also be acknowledged in an obvious attempt to include a wider dietary assessment but this is speculation from a reviewer who shares the opinion that acknowledging such gaps might strengthen the recommendations for future research practice.*
**Response:**

Thank you very much and we agree with your comment. We have inserted the sentence about recall bias associated with 30-day recall (line number 168- 169).

**Comment 4**: *Line 267- Elaborate on the inclusion of pregnant women taking Fansidar (line 267,278, 336), iron and folic acid (line270) (chemicals that the reviewer considers to be medication) when the exclusion criteria excluded women taking medication.*
**Response**: Thank you very much for pointing this out and, we have added information to clarify the inclusion of pregnant women who are taking Fansidar (line number 129-130).

**Comment 5:** *285- The discussion appears to underreport the relationship between the socioeconomic environment or disease states like anemia, hypoalbuminemia etc to dietary intake even though such indices were reported to have been captured in the method and results. As a reader i remain unquenched as to whether the relationship was not reported because the relationship was insignificant to report or it was simple neglect..*

**Response:** Thank you very much for pointing this out. After controlling for the cofounders and effect modifiers, we could not establish a significant relationship between the socioeconomic, environment or disease states with dietary intake.

**Reviewer #2
Comment 1:** *We do not assess poor dietary intake, rather dietary intake to ascertain if its poor or not*
**Response:** Thank you very much and we agree with your comment. We have made that correction throughout the manuscript.

**Comment 2**: *in the methods: concern on authors assessing {“poor diet”) poor diet is an outcome of dietary assessment. You can only determine of the respondents consumed poor diet or good diet after the assessment. It is a product of assessment. I am not sure there is a tool designed to assess poor diet. We have tools to assess dietary intake. Analysis will confirm if the diet was poor or not. If the authors have to focus on poor diets, then they need to state of there are other findings that poor diets in the study population that has led to this study to assess the factors contributing to poor intake. However, if there is no primary data on this, then the focus should be to assess dietary intake.*

**Response**: We highly appreciate the insightful comments and, we have made the correction as suggested.

**Comment 3**: *There is need to review the statements in the whole manuscript, e.g. the statement “A cross-sectional study design was conducted in Mbeya” should delete the word design*

**Response**: Thank you very much for pointing this out and, we have deleted the word ‘design’ as suggested.

**Comment 4**: *The refined grains are portrayed as a good thing while in essence the focus of assessment should be the proportion of mothers consuming unrefined grains.*

**Response:** Thank you very much and we agree with your comments. We have rephrased the paragraph accordingly (line number 271-282).

**Comment 5:** *Poor dietary intake has seriously detrimental consequences for pregnancy and born children both in developed and developing countries (this statement has been overused, in the abstract, introduction and discussions)*

**Response:** Thank you very much and we agreed with the comments and made corrections as suggested both in the abstract, introduction, and discussions.

**Comment 6**: *In the discussion, the comparison between pregnant women and women with HIV may not be the ideal. Persons with HIV tend to receive a lot of nutrition education to improve their nutrition status given the effects of poor nutrition on HIV outcomes.*

**Response:** Thank you very much for pointing this out. For clarity, we deleted the words ‘HIV-negative’ (line number 311).

**Comment 7**: *The findings and the title need to be aligned better; the results have had very specific association cohabiting, obesity, and malaria treatment. Maybe the title should consider capturing this. From the findings cohabiting did promote good dietary intake, it therefore does not qualify to be a factor of poor dietary intake.*

**Response:** Thank you very much and we agree with your comment. In the appropriate areas, the word ‘poor’ was deleted.

**Comment 8:** *The title suggests factors associated with poor dietary intake. Since the title has a bias on the poor intake, then emphasis on this should be discussed. It would be good to highlight possible factors that make cohabiting promote good dietary intake, this is not clear.*

**Response:** We agree with your comment and made changes as suggested.

**Comment 9**: *I also note only 5 % of the women were obese/overweight, but that’s also quite a small proportion and wondering if this is sufficient to draw the conclusion on effects of obesity on poor dietary intake. What is the link, a review of more literature for the discussions.*

**Response:** Thank you very much and we agreed with comments. We have re-written the sentences (line number 340-342 and 352-353).

**Comment 10:** *Fansider intake seems to have promoted dietary intake, however authors should discuss why this is so. How does malaria treatment improve dietary intake?*
**Response:** Thank you very much and we agreed with comments. We have re-written the paragraph for the clarity (line number 357-362).

**Comment 11**: *The only dietary items focused on in the results are vegetables and fruits and partly grains, why haven’t the authors mentioned the other foods? Were they not of interest to pregnant women especially proteins (animal sources) given the nutrient needs in pregnancy? Especially iron?*

**Response:** Thank you very much for pointing this out. All dietary items findings were presented (line number 270-275) and discussed depend on their significance.

**Reviewer #3**

**Comment 1**: *Language editing is required. I started to correct some of the grammatical errors, but it became evident that there are many errors that will require language editing to fix these.*

**Response**: Thank you very much and, we highly appreciate the insightful and helpful comments on our manuscript. We agree with your comment and edited the language.

**Comment 2**: *Dietary intake and diet quality assessment: More detail on the exact process followed to collect dietary intake data is required. Please see manuscript provided for more feedback.*

**Response:** Thank you very much for pointing this out and we have re-checked and addressed small missing parts in the method section.

**Comment 3**: *I am also concerned about the relevance of using a tool developed for coronary heart disease risk when numerous other tools/instruments are available to determine dietary quality in pregnancy.*

**Response:** Thank you very much for pointing this out. The main reason behind selecting this tool is the fact that it has already been used among women of reproductive age in Tanzania (line number 149-151).

**Comment 4**: *Discuss anthropometric measurements under a separate heading. Also, please indicate which standardised techniques and/or references were used for measuring anthropometry.*
**Response**: We have added a sub-section of anthropometric measurements and also indicated citation (line number 193-199).

**Comment 5**: *Data analysis: Please indicate what cut-off level was used when reporting on P-values.*

**Response:** The cut-off level of statistical significance was P-value = 0.05. The arbitrary statistic level of 0.7 was only used for selecting variables for multivariate analysis.

**Comment 6:** *Ethics statement: Please indicate whether informed assent was obtained from minors.*
**Response:** Thank you very much for pointing this out. Informed consent was obtained from minors (pregnant women of age below 18 years).

**Comment 7**: *Dietary intake and diet quality: What was the mean/median PDQS score and how many women in the total sample were classified as having poor PDQS score?*

**Response:** We agreed with your comment. We have added a sentence for median PDQS score and, the total number of pregnant women classified as having poor PDQ score (line number 268-270).

**Comment 8:** *This entire section (lines 258 - 265) needs to be rephrased since the text does not clearly explain what is indicated in Table 2.*

**Response:** We agree with your comment and rephrased the entire section (line number 270-282).

**Comment 9**: *Sentence in lines 261 - 264 needs to be rephrased as it does not make sense currently.*

**Response:** We agree with your comment and rephrased the sentence.

**Comment 10**: *Lines 264 - 265: "Refined grains, baked goods and sugar-sweetened beverages were the most commonly consumed unhealthy foods" - According to the data presented in Table 2, 6.2% of women consumed four servings or more from the sugar sweetened beverages group per week? Thus it does not make sense how you make this statement?*

**Response:** Thank you very much and we agree with your comment and rephrased the sentence.

**Comment 11**: *Bivariate analysis:
Adequately described. Please make sure of P-values, since the P-value for those who received Fansidar tables during pregnancy is indicated as 0.077 in the table, but in the text it is flagged as being significant? It will also be good to explain (either in the introduction or discussion) what these tablets are given for and how often.*

**Response:** We have added more description on P-value cut-off used at the bivariate and multivariate analysis (line number 226-228).

**Comment 12**: *Multivariate analysis: Again, please verify what cut-off level/value was used when reporting on P-values. No P-values indicated in Table 4, thus the reference to P-value in line 284 does not make sense.*

**Response:** We agree with your comment and we have deleted the P-value indicated the line 284.

**Comment 13**: *Discussion: Needs language editing.
Also consider differences on dietary assessment tools used ad possible reasons for differences between findings from the current study and that of others (line 294).*

**Response:** We agree with your comments. We have edited the language throughout the manuscript and add the reasons for differences between findings from the current study and that of others.

**Comment 14**: *Lines 333-344: Regarding IPT needs to be discussed in context of the findings of the current study.*
**Response**: Thank you very much for pointing this out. We have rephrased the paragraph.

Sincerely,

Ray Masumo, PhD
