# Supplementary material for: Dietary intake and associated risk factors among pregnant women in Mbeya, Tanzania
Source: PLOS Glob Public Health. 2024 Jan 5;4(1):e0002529. doi: 10.1371/journal.pgph.0002529 (PMC10769095; doi:10.1371/journal.pgph.0002529)
Supplement: Attachment — Submitted filename: A rebuttal letter2_07_11_2023.docx [file pgph.0002529.s005.docx]

Dear Dickson Abanimi Amugsi, PhD,

Once again, thank you very much for giving us the opportunity to submit a second revised draft of our manuscript titled “Dietary intake and associated risk factors among pregnant women in Mbeya, Tanzania” to *PLOS Global Public Health*.

We appreciate the time and effort that you and reviewers have dedicated to providing valuable feedback on our manuscript.

Here is a point-by-point response to the journal requirement and reviewers’ comments and concerns.

**Journal Requirement**

**Comment 1:** *Please review your reference list to ensure that it is complete and correct. If you have cited papers that have been retracted, please include the rationale for doing so in the manuscript text, or remove these references and replace them with relevant current references. Any changes to the reference list should be mentioned in the rebuttal letter that accompanies your revised manuscript. If you need to cite a retracted article, indicate the article’s retracted status in the References list and also include a citation and full reference for the retraction notice*

**Response**: We highly appreciate this comment and, we have carefully reviewed the reference list. Reference number 37 ‘World Health Organisation, Food Based Dietary Guidelines in the WHO European Region. 2003. URL: http://www.euro.who.int (Accessed June 2023)’ is retracted and, now reference number 37 is ‘World Health Organization. Comparative analysis of nutrition policies in the WHO European Region: a comparative analysis of nutrition policies and plans of action in WHO European Region. World Health Organization. Regional Office for Europe.2006’ (line number 538-540).

**Review Comments to the Author**

**Reviewer #1**

**Comment 1:** *Line267-I remain unanswered by the lack of a rationale or clarity towards the reported exclusion of pregnant women taking medication whilst there was inclusion of pregnant women taking fansidar, iron supplements etc which by definition can fall under the category of medication. Kindly clarify this antagonistic exclusion criterion.*

**Response:** Thank you very much for this comment, the statement on ‘*the* *exclusion criteria of pregnant women taking medication…..’* has been rewritten and now read as ‘Pregnant women who refused to consent or who were unable to communicate due to illness were excluded from this study’ (line number 125-126).

**Reviewer #3**

**Comment 1:** *Lines 281 - 282: Please revise this sentence and rephrase to perhaps read as follows: "However, refined grains and baked goods represented the highest percentage of servings consumed per week out of the six unhealthy foods assessed."*

**Response:** Thank you very much for this comment and, we carefully rephrased the sentence as suggested (line number 270-271).

**Comment 2:** *Lines 316 - 319: Please revise this sentence and consider rephrasing to: "Guidelines of other countries like UK and USA recommend a plant-based diet, rich in fruit, vegetables...."*

**Response:** We highly appreciate the reviewers’ insightful comment. We have rephrased the sentence accordingly (line number 303-304).

**Comment 3:** *Line 349: the word "Finding" should rather be "Findings"*

**Response**: Thank you very much and, we have made the correction as suggested (line number 335).

**Comment 4**: *Line 353: The word "obese" should rather be "obesity"*

**Response:** Thank you very much for pointing this out. We have corrected the word ‘obese’ to ‘obesity’ throughout the manuscript.

**Comment 5**: *Line 356: Insert the word "an" between the words "highlighted" and "finding"*

**Response**: Thank you very much and, we have made the correction as suggested (line number 340)

Sincerely,

Ray Masumo, PhD
