# Supplementary material for: Dietary intake and associated risk factors among pregnant women in Mbeya, Tanzania
Source: PLOS Glob Public Health. 2024 Jan 5;4(1):e0002529. doi: 10.1371/journal.pgph.0002529 (PMC10769095; doi:10.1371/journal.pgph.0002529)
Supplement: Attachment — Submitted filename: A rebuttal letter2_Kilel_16_11_2023.docx [file pgph.0002529.s006.docx]

Dear Dickson Abanimi Amugsi, PhD,

Once again, thank you very much for giving us the opportunity to submit a second revised draft of our manuscript titled “Dietary intake and associated risk factors among pregnant women in Mbeya, Tanzania” to *PLOS Global Public Health*.

We appreciate the time and effort that you and reviewers have dedicated to providing valuable feedback on our manuscript.

Here is a point-by-point response to the reviewers’ comments and concerns.

**Review Comments to the Author**

**Reviewer #1**

**Comment 1:** *The exclusion criteria remains unchanged, it mentions that pregnant women taking medication were excluded and fails to explain why if this was so, there was inclusion of pregnant women taking fansidar (chemoprophylaxis for malaria), iron and folate supplements. All these chemicals are also medication so this antagonistic statement remains an issue.
Recommendations would advice to provide a meaningful exclusion criteria eg, the study excluded pregnant women taking medication for other reasons except malaria chemoprophylaxis plus iron and folate supplements.*

**Response:** We highly accept your insightful comments and suggestions. We have agreed with your suggestion (line number 125-126).

Sincerely,

Ray Masumo, PhD
